# Does Temperature Affect COVID-19 Transmission?

**DOI:** 10.3389/fpubh.2020.554964

**Published:** 2020-12-22

**Authors:** Aly Zein Elabdeen Kassem

**Affiliations:** Agricultural Marketing and Information Systems Department, Center of Agricultural Planning and Development, Institute of National Planning, Cairo, Egypt

**Keywords:** COVID-19, SARS-CoV-2, infectious disease, COVID-19 transmission, temperature

## Abstract

This study utilizes the non-linear least squares method to estimate the impact of temperature on COVID-19 cases per million in forty-three countries, divided into three groups as follows: the first group is composed of thirteen countries that announced the first COVID-19 cases in January 2020, while the second and third groups contain thirteen and seventeen countries, respectively, that witnessed the pandemic for the first time in February and March of the same year. This relationship was measured after four time periods from the date of reporting the first case until April 1, April 15, May 15, and July 8, 2020. The results show an inverse relationship between COVID-19 cases per million and the temperature in the studies of the four-time periods for the three-country groups. These results were only significant statistically (*p* < 0.1) after 110.8, 164.8 days on average from the beginning of the pandemic in the case of “January” countries.

## Introduction

There is no doubt that the containment of the COVID-19 pandemic, caused by the emerging coronavirus (SARS-CoV-2), is currently the primary concern worldwide. The characteristics of this pandemic made it difficult even for the most advanced health systems to control it after it started in Wuhan, China in late 2019.

COVID-19 has spread into most countries of the world due to its extremely high transmission rate of 2–2.5 (1). The number of COVID-19 cases globally as of July 8, 2020, at 08:24 GMT, was 11,965,661, of which 57.78% recovered, 4.57% died, and 37.65% were still active. According to official statistics, China, the source of the pandemic, managed to close 93.99% of cases with recovery, 5.54% with deaths (three cases per one million), and only 0.47% of cases were still active. While the US topped the list of the most affected countries, with a case number of about 3,097,417, representing 25.89% of global cases of which, 51.93% cases were still active. Furthermore, the combined COVID-19 cases in the US, Brazil, India and Russia amounted to 51.96% of the global cases during the study time. (https://www.worldometers.info/coronavirus/).

It is noteworthy that COVID-19 cases are mostly concentrated in the central and northern areas in the affected countries that represent capitals, densely populated cities, economic and financial centers, especially in the developed countries. For instance, New York, the US economic capital in the far northeast, was one of the most affected States. Likewise, the province of Lampodria in northern Italy, responsible for 40% of the industrial production (2), was the most affected area in Italy. Furthermore, COVID-19 has swept through Madrid, the Spanish capital, and the most important financial and economic center (3). In addition, the pandemic was concentrated in Wuhan, the transportation and industry hub in central China. In Germany, the province of Bavaria, the second-largest German city, in terms of population and the producer of 18% of the gross German domestic production (4) was the most affected. As for France, the pandemic targeted the north-central region, Ile-de-France, the richest and the most important French and European region in terms of research, development, and innovation (5).

Coronaviruses are large, enveloped RNA viruses of both medical and veterinary importance (6). The envelope structures of SARS-CoV-2 are sensitive to physical and chemical conditions and can be destabilized or damaged by heat, ultraviolet (UV) light or extreme pH (7). The outermost structural protein of the SARS-CoV2 “Spike protein” showed active and inactive states at different temperatures (8). In such a way, regions that have low temperatures are more prone to infection than those with higher temperatures (9). The COVID-19 cases increased toward the Earth's poles with increasing latitude (7). Accordingly, coronavirus peaks occur in winter, taking the form of local epidemics that last a few weeks or months (10).

Several studies indicate that the transmission of COVID-19 is affected by temperature. An inverse correlation was found between temperature and the daily number of infections (11–17). Other studies determine the temperature range for this effect. For instance, the virus transmission is hindered by specific humidity above 6 g/kg and mean air temperature above 11°C (18). COVID-19 can be seasonal with the optimal temperature range of 5°C−14 and the peak of 10°C (19). Another study estimates that every 1°C increase in the minimum temperature leads to a decrease in the cumulative number of cases by 0.86 (20). In contrast, other studies deny or underestimate the effect of temperature on COVID-19 (21–25). It is indisputable that some of these results were affected by the methodology of analysis used, the countries chosen to carry out the study, and the other confounding factors that affect the phenomenon that may not have been neutralized in some of these studies.

Not only are climate and meteorological factors expected to affect the transmission of COVID-19 (17, 26), but there are also many other variables, i.e., social distancing, age, GDP per capita, ethnicities, health, poverty, diabetes, coronary heart disease, physical inactivity, alcohol consumption, tobacco abuse, and access to primary care (27). This paper investigates the impact of temperature on COVID-19 transmission, represented in cases per million.

## Methods

The transmission rate of COVID-19 is expressed as the daily number of infections (11, 25, 28), or the total number of confirmed cases (7, 9, 12, 16, 23). In other studies, the number of cases accumulated over a period of time (18, 20, 29), average daily cumulative rate of confirmed cases (13), or cases per 100000 (27) represent COVID-19 transmission. In addition, the virus spread is indicated as the growth rate of the confirmed cases (21, 24, 30), the effective reproductive number of infection (22), or the doubling time of the confirmed cases number (26). Others use cases per 1-km^2^ (15).

In some studies, the temperature is expressed as the average daily temperature (13, 14, 20, 22, 25, 29), or the average temperature over a period of time (7, 9, 23). Others use the 14-day exponential moving averages (EMAs) of daily average temperature (28). For this study, the average temperature over a period of time is used. Countries are represented in terms of temperature by the most affected cities, or by capitals.

The non-linear least-squares method is employed to estimate the relationship between COVID-19 transmission and temperature using the STATA statistical software package (version 16.1; StataCorp LLC). The exponential function was suggested in Equation (11) to represent the relationship between the number of COVID-19 cases per million as a dependent variable (y), and the average temperature as an independent variable (x) (31).

(1) $$\begin{array}{l} y_{it}=\alpha e^{\beta Xit} \end{array}$$

Where:

yit: is COVID-19 per million in country “i” at the end of the period “t”

α, β: is the model parameters.

xit: is the average temperature in the country “i” during the period “t.”

Obtaining the natural logarithm of both sides of Equation (1), the following equivalent equation can be obtained:

(2) $$\begin{array}{l} {ln\;y}_{it}=\text{ln }\alpha +\beta x_{it} \end{array}$$

Where it was possible by converting to Equation (2) to obtain a formula for a linear regression model to which, the error component ε can be added to become as follows:

(3) $$\begin{array}{l} {y^{\prime }}_{it}=\alpha^{\prime }+\beta x_{it}+\;\varepsilon \end{array}$$

This study assumes that the prevalence of COVID-19 increases as temperature decreases and vice versa. Hence, the main hypotheses are:

H0: there is no inverse relationship between COVID-19 per million and the temperature.
H1: there is an inverse relationship between COVID-19 per million and the temperature.

Given the low number of observations here, a level of significance (*p* < 0.1) has been adopted (32). Data on COVID-19 cases per million (Supplementary Tables 3, 7, 11) and temperature (Supplementary Tables 4, 8, 12) in forty-three countries were collected. These countries were divided into three groups, as follows: the first group consists of thirteen countries, eleven of which have experienced the pandemic for the first time in the last third of January 2020, namely: Australia, Finland, France, Germany, Italy, Malaysia, Russia, Spain, Sweden, UK, and the US, in addition to Japan and South Korea that witnessed the pandemic in the second third of the same month. The second group consists of thirteen countries, all of which have reported the first case of COVID-19 in the last third of February 2020, namely: Armenia, Austria, Belarus, Croatia, Czechia, Denmark, Estonia, Ireland, Lithuania, New Zealand, Norway, Romania, and Switzerland. Finally, the last group consists of seventeen countries, all of which have experienced the COVID-19 pandemic during the first week of March 2020, namely: Albania, Bosnia and Herzegovina, Chile, Jordan, Moldova, Morocco, Paraguay, Peru, Poland, Portugal, Saudi Arabia, Serbia, Slovakia, Slovenia, Tunisia, Turkey, and Ukraine.

For more reliable results, comparable countries were intentionally chosen in the same group (Supplementary Tables 1, 5, 9). Furthermore, the primary comparison criterion was the extent to which the country succeeded in closing nearly half or more of the COVID-19 cases, accompanied by a decrease in the death rate attributed to the cases that were closed. This may indicate the status of the country's health system, as well as other sub-criterions considered, such as the number of tests per million as an indicator of spending on health in the country. It was also taken into account that there would not be a large disparity in the population, and that is why Brazil and Pakistan were excluded from the “February” group, for example. It was also taken into consideration that there was no disparity in the population density. Nevertheless, there were some necessary exceptions, such as the inclusion of France, Italy, and Spain in the “January” group, despite their high death rates, compared to the other group's members. However, they were included as a result of their similarity to other group's countries, in terms of their ability to close more than half of COVID-19 cases, as well as having high health spending, expressed in the number of tests per million. They also have a high median age as with the rest of the group - except Malaysia and Australia.

This relationship between the study's variables was measured for each group after four-time periods from the date of the first case reported until April 1, April 15, May 15, and July 8, 2020, respectively (Supplementary Tables 2, 6, 10).

For ensuring the validity of the results, Cook's distance and DFFITS tests were performed to show the influence of each observation on the fitted response values. The goodness of fit of the model parameters was checked by these two methods in which, outliers, leverage, and influential observations that affect the values of the fitted parameters were omitted (33).

Data on COVID-19, analyzed in this paper, was collected from one website (https://www.worldometers.info/), which provides global COVID-19 live statistics. The website is independent and is frequently cited as a source in journal articles. It was also voted as one of the best free reference websites by the American Library Association (http://www.ala.org/rusa/). As well, (https://www.timeanddate.com/) was utilized to obtain the monthly temperature in the most affected cities during the study's four-time periods. The site helps obtain the average monthly temperature directly without further calculations.

## Results

The relationship parameters between COVID-19 cases per million in the three studies' groups of countries and the average temperatures are estimated as follows (Table 1).

**Table 1:** Results of the regression models.

**Months**	**Days^a^**	**Actual No. of obs^b^**	**${\text{R}}_{\text{adj}}^{2}$**	**F**	**Pro. > F**	**Intercept (S.E.)**	***P*-value**	**Temp. (X) (S.E.)**	***P*-value**
January	68.8	10	−0.1197	0.04	0.8503	6.367 (0.489)	0.000	−0.010 (0.050)	0.85
	80.8	11	0.0005	1.01	0.3422	7.325 (0.491)	0.000	−0.037 (0.037)	0.342
	110.8	12	0.2261	4.21	0.0672	8.298 (0.574)	0.000	−0.089 (0.043)	0.067^*^
	164.8	12	0.2356	4.39	0.0626	9.318 (0.862)	0.000	−0.121 (0.058)	0.063^*^
February	35.2	11	0.0508	1.53	0.2472	6.882 (0.712)	0.000	−0.151 (0.122)	0.247
	49.2	11	−0.0941	0.14	0.7171	6.825 (0.666)	0.000	−0.034 (0.090)	0.717
	79.2	10	0.0647	1.62	0.2384	8.130 (0.706)	0.000	−0.094 (0.074)	0.238
	133.2	11	−0.0424	0.59	0.4608	8.611 (1.369s)	0.000	−0.083 (0.108)	0.461
March	28.35	14	0.1001	2.45	0.1438	4.994 (0.495)	0.000	−0.056 (0.036)	0.144
	42.35	15	−0.0453	0.39	0.5412	5.690 (0.647)	0.000	−0.028 (0.044)	0.541
	72.35	13	−0.0722	0.19	0.6701	7.043 (1.396)	0.000	−0.042 (0.103)	0.67
	126.35	13	−0.0908	0	0.9702	7.317 (2.086)	0.005	−0.005 (0.122)	0.97

a *Days from the 1st case reported*.

b *After excluding outliers, leverage and influencer observations*.

* *Significant at 10% significance level*.

**Figure 1:**
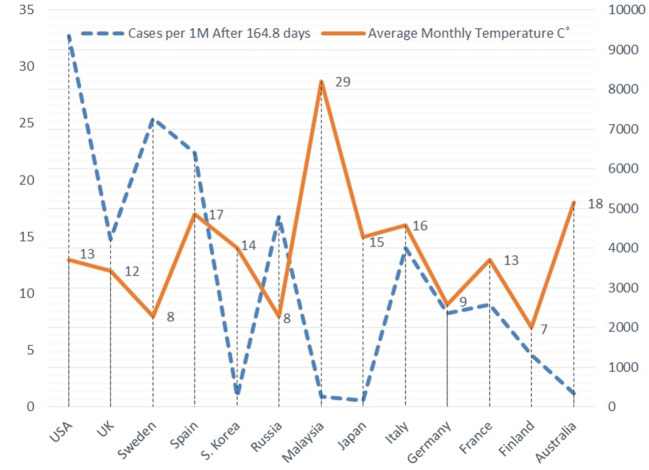
COVID-19 cases per 1 M and temperature after 164.8 on average in January group countries.

The results show an inverse relationship between the study's variables in the three groups of countries under study, in all the four-time periods since the first case was reported. The null hypothesis was rejected in favor of the alternative hypothesis at (*p* < 0.1) only after 110.8 and 164.8 days on average from the first case reported in the “January” group countries. Figure 1 illustrates an example of the inverse relationship between cases per one million and weather temperature after 164.8 days in average, in the case of the “January” countries group.

By substituting the regression parameters for the “January” group countries “after 110.8 and 164.8 days” in Equation 3, Equations 4 and 5 can be obtained:

(4) $$\begin{array}{l} lny_{it}=8.298-0.089\;x_{it} \end{array}$$

(5) $$\begin{array}{l} lny_{it}=9.318-0.121\;x_{it} \end{array}$$

Applying e to both sides:

(6) $$\begin{array}{l} y_{it}={\left(e^{-0.089}\right)}^{x_{it}}+e^{8.298}=EXP{\left(-0.089\right)}^{x_{it}}\\ \;+EXP\left(8.298\right) \end{array}$$

(7) $$\begin{array}{l} y_{it}={\left(e^{-0.121}\right)}^{x_{it}}+e^{9.318}=EXP{\left(-0.121\right)}^{x_{it}}\\ \;+EXP\left(9.318\right) \end{array}$$

Equations 6 and 7 are used for predicting the development of COVID-19 cases per million, in terms of weather temperature (Supplementary Tables 13, 14).

## Discussion

Although temperature is one of the factors that influence COVID-19 prevalence, there are other important factors that have worsened the situation in countries that were heavily invaded by the pandemic, such as the US, Spain, and Italy. Perhaps those countries were relatively late in imposing precautionary measures, unlike other similar countries, in terms of temperature at that time, such as China, South Korea, and Japan that managed to flatten the curve of new cases of COVID-19. In addition, the later countries utilized distinguished mechanisms of early mitigation measures well, including the big data techniques to contain the pandemic from its springs. Therefore, this was evidenced by the determination coefficient (R bar squared) in regression models (Table 1), which was 23.56% at most, indicating that 76.44% of the phenomenon is explained by other factors.

The preprint-results of this study (34) related the negative relationship between COVID-19 cases per million and temperature to the number of days since the first case was reported. Although temperature affects COVID-19 transmission in its early stages, cases per million reach a critical mass after the successive exponential increase, and temperature no longer has a significant influence on the pandemic transmission. As for this study, it coincides with the preprint's results, in terms of the direction of the relationship between the study variables. In contrast, the effect of temperature on the prevalence of COVID-19 was not statistically confirmed here in ten out of twelve observations.

Perhaps, the preprint's results were affected by the crude comparison between incomparable countries, in terms of case ascertainment, connections between the country and the affected areas, population density, applied control measures to the country, and timing at which they were instituted. Regardless of the difficulties that concern the availability of a sufficient number of countries that would be compared, this study tries to include more comparable countries as much as possible. In addition, the preprint's results compared the relationship between the study variables after only two periods for two different groups of countries (72 days in the case of January countries, and after 44 days in the case of February countries), whereas this study intentionally deepens the analysis to estimate the relationship in four periods for each of the three groups of countries under study.

Referring to Supplementary Table 13, it turns out from Table 2 that the observed COVID-19 cases per million after 110.8 days in average, from the first case, reported in France, Italy, Spain, Sweden, UK, and the US, were higher than its expected values, in respect to the average temperature. On the contrary, the observed COVID-19 cases per million, with regards to the temperature in Australia, Finland, Germany, Japan, Malaysia, Russia, and South Korea, were lower than expected after the same period. Likewise, the same group of countries showed almost identical behavior after 164.8 days on average except for Russia, where the numbers of observed COVID-19 cases were greater than expected (Supplementary Table 14, Table 2). The findings of this study assume that the data declared by countries are correct and accurate. But in the case of assumed underestimation or underreporting, actual cases per million in these countries can be expected as in Table 2. The same table show that the observed COVID-19 cases per million were less than their expected values in Japan by about 11 times, and by about 7–7.5 times in South Korea. Contrariwise, the observed COVID-19 cases per million were ~4–5 times more than estimations in Spain, and three times in Italy, whereas, the COVID-19 cases per million observed in the US were twice to four times its estimated values.

**Table 2:** Observed and estimated COVID-19 cases per million, in terms of temperature in the “January-group” countries.

**Countries**	**After 110.8 days on average**			**After 164.8 days on average**		
	**Temp. (C°)**	**COVID-19 cases per 1 M**		**Temp. (C°)**	**COVID-19 cases per 1 M**	
		**Observed**	**Estimated in terms of temp**.		**Observed**	**Estimated in terms of temp**.
Australia	20.4	276	654	18.29	348	1,213
Finland	3.6	1,124	2,915	7.43	1,311	4,526
France	10.2	2,175	1,620	12.57	2,586	2,427
Germany	6.4	2,092	2,272	9.14	2,367	3,678
Italy	13.4	3,702	1,219	16.14	4,002	1,575
Japan	11.6	127	1,430	15	158	1,808
Malaysia	29	212	304	28.71	268	343
Russia	4.2	1,801	2,763	8.43	4,802	4,009
S. Korea	10.2	215	1,620	14.29	272	1,971
Spain	13.4	5,868	1,219	16.86	6,400	1,443
Sweden	5	2,894	2,573	8.43	7,261	4,009
UK	10	3,489	1,649	12	4,218	2,601
USA	8.4	4,445	1,902	12.86	9,357	2,343

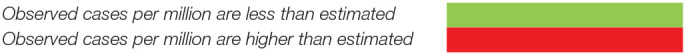

One of the main criteria for selecting the “January” group countries was that they have advanced health systems and are expected to have a high degree of data reliability. Nevertheless, differences have emerged between the estimated and observed values. It is expected that these differences will be greater in the case of countries with less advanced health systems, and less reliable data recording. This was demonstrated when data about Togo and South Africa, instead of Turkey, was included in the “March” countries (Supplementary Tables 15–19). Through this data and using equations as in Supplementary Table 19, prediction tables of COVID-19 cases per million, in terms of temperature were obtained (Supplementary Tables 20, 21). It is revealed from Supplementary Table 22 that COVID-19 cases per million after 28.56 days in average, from the first case reported in Albania, Bosnia and Herzegovina, Chile, Moldova, Peru, Portugal, Saudi Arabia, Serbia, and Slovenia, were higher than its expected values, in terms of temperature. Far from this, the observed COVID-19 cases per million in Jordan, Morocco, Paraguay, Poland, Slovakia, South Africa, Togo, Tunisia, and Ukraine were lower than expected, in light of temperature. In a second period after 42.56 days on average, most of the above-mentioned courtiers showed a relatively large discrepancy between the observed and estimated COVID-19 cases per million (Supplementary Table 22). This large discrepancy may be due to underestimation or underreporting. In addition, this also may be associated with the number of tests per million, performed to detect COVID-19. For instance, it was noted that the estimated COVID-19 cases in Portugal that performed 124,698 tests per million were approximately twice the observed figures only. On the other hand, the estimated COVID-19 cases in Togo, which performed 4,025 tests per million at best, were 82 times the observed cases.

Finally, it is highly recommended that the relationship between COVID-19 cases per million and the temperature should be estimated at different time periods thereafter; in order to monitor the phenomenon, either in later stages or earlier than those observed in this study.

## Conclusion

All the findings reached are presented in this study, including those introduced by the pre-print, or the attempts to replace some countries in the third group (Supplementary Tables 19–22). All the findings agree that the relationship between the temperature and the transmission of COVID-19 has an opposite direction, despite the variation in the level of significance, from being significant to insignificant. The differences in the criteria of selecting countries may lead to the variation in the statistical significance magnitude, but they have not affected the direction of the relationship. This study reports that the relationship between COVID-19 transmission and temperature is marginally and statistically confirmed (*p* < 0.1) in just two observations out of twelve. This may indicate that factors other than temperature are the most influential on the transmission of COVID-19.

## Data Availability Statement

The raw data supporting the conclusions of this article will be made available by the authors, without undue reservation.

## Author Contributions

The author confirms being the sole contributor of this work and has approved it for publication.

## Conflict of Interest

The author declares that the research was conducted in the absence of any commercial or financial relationships that could be construed as a potential conflict of interest.
