# Supplementary material for: Does Temperature Affect COVID-19 Transmission?
Source: Front Public Health. 2020 Dec 22;8:554964. doi: 10.3389/fpubh.2020.554964 (PMC7793668; doi:10.3389/fpubh.2020.554964)
Supplement: Supplementary file 1 [file Data_Sheet_1.PDF]

Table S1: Statistics of "January" comparable Countries.

Country	(1) Land* area (Km2)	(2) Population**	Population density (people/km2) =(2)/(1)	Median*** age	(3) Total** cases (July 8th)	(4) Total** death (July 8th)	(5) Total recovered*** (July 8th)	(6) closed cases (July 8th) =(4)+(5)	% closed cases (July 8th) =(6)/(3)	% deaths (July 8th) =(4)/(6)	Cases per million*** (July 8th)	tests per 1 million** (July 8th)
Australia	7,682,300	25,534,483	3.324	37.5	8,886	106	7,487	7,593	85.45%	1.40%	348	111,875
Finland	303,890	5,539,283	18.228	42.8	7,262	329	6,700	7,029	96.79%	4.68%	1,311	46,112
France	547,557	65,278,422	119.218	41.7	168,810	29,933	77,655	107,588	63.73%	27.82%	2,586	21,212
Germany	348,560	83,800,169	240.418	47.8	198,355	9,103	182,700	191,803	96.70%	4.75%	2,367	70,099
Italy	294,140	60,458,771	205.544	46.5	241,956	34,899	192,815	227,714	94.11%	15.33%	4,002	94,338
Japan	364,555	126,462,025	346.894	48.6	19,981	978	17,197	18,175	90.96%	5.38%	158	4,033
Malaysia	328,550	32,365,672	98.511	29.2	8,674	121	8,481	8,602	99.17%	1.41%	268	25,210
Russia	16,376,870	145,937,526	8.911	40.3	700,792	10,667	472,511	483,178	68.95%	2.21%	4,802	149,317
S. Korea	97,230	48,691,176	500.783	43.2	13,244	285	11,970	12,255	92.53%	2.33%	272	29,272
Spain	498,800	46,751,563	93.728	43.9	299,210	28,392	NA	NA	NA	NA	6,400	122,652
Sweden	410,340	10,101,088	24.616	41.1	73,344	5,447	NA	NA	NA	NA	7,261	51,503
UK	241,930	67,887,387	280.608	40.6	286,349	44,391	NA	NA	NA	NA	4,218	205,782
USA	9,147,420	331,026,718	36.188	38.5	3,097,417	133,977	1,355,081	1,489,058	48.07%	9.00%	9,357	117,217
Average	2818626.3	80756483.3	152.1	41.7	394175.4	22971.4	233259.7	255299.5	83.65%	7.43%	3334.6	80663.2
Minimum	97230.0	5539283.0	3.3	29.2	7262.0	106.0	6700.0	7029.0	48.07%	1.40%	158.0	4033.0
Maximum	16376870.0	331026718.0	500.8	48.6	3097417.0	133977.0	1355081.0	1489058.0	99.17%	27.82%	9357.0	205782.0

* https://www.worldometers.info/geography/largest-countries-in-the-world/ (Last accessed July 8th, 2020) ** https://www.worldometers.info/coronavirus/ (Last accessed July 8th, 2020; 08:24 GMT). *** https://www.citypopulation.de/en/world/bymap/MedianAge.html (Last accessed July 8th, 2020).

Table S2: 1st case reported date, and the four time periods of the study for "January" countries

Country	1st case reported(1)	period1*	period2**	period3***	period4****
Australia	24/01/2020	68	82	112	166
Finland	28/01/2020	64	78	108	162
France	23/01/2020	69	83	113	167
Germany	26/01/2020	66	80	110	164
Italy	29/01/2020	63	77	107	161
Japan	14/01/2020	78	92	122	176
Malaysia	24/01/2020	68	82	112	166
Russia	30/01/2020	62	76	106	160
S. Korea	19/01/2020	73	87	117	171
Spain	30/01/2020	62	76	106	160
Sweden	30/01/2020	62	76	106	160
UK	30/01/2020	62	76	106	160
USA	20/01/2020	72	86	116	170
Average		66.8	80.8	110.8	164.8
Minimum		62.0	76.0	106.0	160.0
Maximum		78.0	92.0	122.0	176.0

(1) https://www.worldometers.info/coronavirus/ (Last accessed April 1st, 2020; 00:02 GMT).

* From 1st case reported till April 1st, 2020. ** From 1st case reported till April 15th, 2020. *** From 1st case reported till May 15th, 2020. **** From 1st case reported till July 8th, 2020.

Table S3: absolute and logarithmic values of the dependent variable for "January" countries, cases per million (y), at the four periods of the study.

Country	y1*	lny1	y2**	lny2	y3***	lny3	y4****	lny4
Australia	187	5.23	253	5.53	276	5.62	348	5.85
Finland	256	5.55	584	6.37	1,124	7.02	1,311	7.18
France*****	799	6.68	1627	7.39	2,175	7.68	2,586	7.86
Germany	857	6.75	1,578	7.36	2,092	7.65	2,367	7.77
Italy	1,750	7.47	2,687	7.9	3,702	8.22	4,002	8.29
Japan	15	2.71	64	4.16	127	4.84	158	5.06
Malaysia	85	4.44	499	6.21	212	5.36	268	5.59
Russia	16	2.77	168	5.12	1,801	7.5	4,802	8.48
S. Korea	191	5.25	207	5.33	215	5.37	272	5.61
Spain	2,052	7.63	3,799	8.24	5,868	8.68	6,400	8.76
Sweden	439	6.08	1,181	7.07	2,894	7.97	7,261	8.89
UK	370	5.91	1,383	7.23	3,489	8.16	4,218	8.35
USA	566	6.34	1,856	7.53	4,445	8.4	9,357	9.14

* https://www.worldometers.info/coronavirus/ (Last accessed April 1st, 2020; 00:02 GMT). ((https://www.worldometers.info/coronavirus/ (Last accessed April 15st, 2020; 12:36 GMT). ***https://www.worldometers.info/coronavirus/ (Last accessed May 15st, 2020; 18:08 GMT). **** https://www.worldometers.info/coronavirus/ (Last accessed July 8th, 2020; 08:24 GMT).

***** France data was adjusted according to the French Public Health Agency

Table S4: Weather temperature (C°) in "January" countries during the study periods.

Country	Most effected cites/ capitals	Jan.	Feb.	Mar.	Apr.	May	Jun.	Jul.	temp1*	temp2**	temp3***	temp4****
Australia	New south wales; Sydney	24	23	21	19	15	13	13	22.67	21.75	20.4	18.29
Finland	Uusimaa	2	1	2	4	9	18	16	1.67	2.25	3.6	7.43
France	Il de France; Paris	6	8	8	14	15	18	19	7.33	9	10.2	12.57
Germany	Munich, Bavaria	2	5	4	10	11	15	17	3.67	5.25	6.4	9.14
Italy	Lombardy, Milan	4	8	20	15	20	22	24	10.67	11.75	13.4	16.14
Japan	Kanto; Tokyo	7	8	11	13	19	23	24	8.67	9.75	11.6	15
Malaysia	Selangor, Shah Alam	29	29	29	29	29	28	28	29	29	29	28.71
Russia	Moscow	0	0	4	5	12	19	19	1.33	2.25	4.2	8.43
S. Korea	Daegu Metropolitan City	4	5	10	12	20	25	24	6.33	7.75	10.2	14.29
Spain	Madrid	8	13	12	14	20	23	28	11	11.75	13.4	16.86
Sweden	Stockholm	4	2	3	7	9	18	16	3	4	5	8.43
UK	London	7	8	8	12	15	17	17	7.67	8.75	10	12
USA	New York	4	5	8	10	15	23	25	5.67	6.75	8.4	12.86

Source: https://www.timeanddate.com/

*the average monthly temperature during the 1st period of the study, equals to the average monthly temperature of January, February and March, 2020. ** The average monthly temperature during the 2nd period of the study, calculated as the average monthly temperature from January to April, 2020. *** The average monthly temperature during the 3rd period of the study, calculated as the average monthly temperature from January to May, 2020. **** The average monthly temperature during the 4th period of the study, calculated as the average monthly temperature from January to July, 2020.

Table S5: Statistics of "February" Comparable Countries.

Country	(1) Land* area (Km2)	(2) Population**	Population density (people/km2) =(2)/(1)	Median*** age	(3) Total** cases (July 8th)	(4) Total** death (July 8th)	(5) Total recovered*** (July 8th)	(6) closed cases (July 8th) =(4)+(5)	% closed cases (July 8th) =(6)/(3)	% deaths (July 8th) =(4)/(6)	Cases per million*** (July 8th)	tests per 1 million** (July 8th)
Armenia	28470	2963331.015	104.0861	36.6	29,820	521	17,427	17,948	60.19%	2.90%	10,063	42,914
Austria	82409	9007823.961	109.3063	44.5	18421	706	16686	17392	94.41%	4.06%	2045	74186
Belarus	202910	9449726.857	46.57103	40.9	64,003	436	51,902	52,338	81.77%	0.83%	6,773	113,685
Croatia	55960	4105395.232	73.36303	43.9	3,272	113	2,229	2,342	71.58%	4.82%	797	21,161
Czechia	77240	10713682.43	138.7064	43.3	12,685	351	7,910	8,261	65.12%	4.25%	1,184	53,670
Denmark	42430	5792359.551	136.5157	42	12,888	609	11,983	12,592	97.70%	4.84%	2,225	199,841
Estonia	42390	1326490.066	31.29252	43.7	2,003	69	1,882	1,951	97.40%	3.54%	1,510	83,338
Ireland	68890	4938696.577	71.6896	37.8	25,538	1,742	23,364	25,106	98.31%	6.94%	5,171	95,972
Lithuania	62674	2722466.96	43.43854	44.5	1854	79	1552	1631	87.97%	4.84%	681	164630
New Zealand	33720	5006514.658	148.4732	37.2	1,537	22	1,492	1,514	98.50%	1.45%	307	83,776
Norway	365268	5422424.242	14.84506	39.5	8,947	251	8,138	8,389	93.76%	2.99%	1,650	66,060
Romania	230170	19233766.23	83.56331	42.5	29,620	1,799	20,534	22,333	75.40%	8.06%	1,540	40,726
Switzerland	39516	8654812.834	219.0205	42.7	32369	1966	29300	31266	96.59%	6.29%	3740	74168
Average	102,465	6,872,115	94	41	18,689	666	14,954	15,620	0.86	0.04	2,899	85,702
Minimum	28,470	1,326,490	15	37	1,537	22	1,492	1,514	0.60	0.01	307	21,161
Maximum	365,268	19,233,766	219	45	64,003	1,966	51,902	52,338	0.99	0.08	10,063	199,841

* https://www.worldometers.info/geography/largest-countries-in-the-world/ (Last accessed July 8th, 2020) ** https://www.worldometers.info/coronavirus/ (Last accessed July 8th, 2020; 08:24 GMT). *** https://www.citypopulation.de/en/world/bymap/MedianAge.html (Last accessed July 8th, 2020).

Table S6: 1st case reported date, and the four time periods of the study for "February" countries

Country	1st case reported(1)	period1*	period2**	period3***	period4****
Armenia	29/02/2020	32	46	76	130
Austria	24/02/2020	37	51	81	135
Belarus	27/02/2020	34	48	78	132
Croatia	21/02/2020	40	54	84	138
Czechia	29/02/2020	32	46	76	130
Denmark	26/02/2020	35	49	79	133
Estonia	26/02/2020	35	49	79	133
Ireland	28/02/2020	33	47	77	131
Lithuania	27/02/2020	34	48	78	132
New Zealand	27/02/2020	34	48	78	132
Norway	25/02/2020	36	50	80	134
Romania	21/02/2020	40	54	84	138
Switzerland	24/02/2020	37	51	81	135
**Average**		35.2	49.2	79.2	133.2
Minimum		32	46	76	130
Maximum		40	54	84	138

(1) https://www.worldometers.info/coronavirus/ (Last accessed April 1st, 2020; 00:02 GMT).

* From 1st case reported till April 1st, 2020. ** From 1st case reported till April 15th, 2020. *** From 1st case reported till May 15th, 2020. **** From 1st case reported till July 8th, 2020.

Table S7: absolute and logarithmic values of the dependent variable for "February" countries, cases per million (y), at the four periods of the study.

Country	y1*	lny1	y2**	lny2	y3***	lny3	y4****	lny4
Armenia	180	5.19	375	5.93	1,365	7.22	10,063	9.22
Austria	1130	7.03	1587	7.37	1790	7.49	2045	7.62
Belarus	16	2.77	395	5.98	2934	7.98	6773	8.82
Croatia	211	5.35	424	6.05	541	6.29	797	6.68
Czechia	309	5.73	574	6.35	782	6.66	1184	7.08
Denmark	494	6.2	1153	7.05	1864	7.53	2225	7.71
Estonia	562	6.33	1055	6.96	1331	7.19	1510	7.32
Ireland	655	6.48	2325	7.75	4859	8.49	5171	8.55
Lithuania	197	5.28	401	5.99	559	6.33	681	6.52
New Zealand	134	4.9	287	5.66	311	5.74	307	5.73
Norway	856	6.75	1233	7.12	1515	7.32	1650	7.41
Romania	117	4.76	375	5.93	854	6.75	1,540	7.34
Switzerland	1,919	7.56	3043	8.02	3529	8.17	3740	8.23

* https://www.worldometers.info/coronavirus/ (Last accessed April 1st, 2020; 00:02 GMT). ((https://www.worldometers.info/coronavirus/ (Last accessed April 15st, 2020; 12:36 GMT). ***https://www.worldometers.info/coronavirus/ (Last accessed May 15st, 2020; 18:08 GMT). **** https://www.worldometers.info/coronavirus/ (Last accessed July 8th, 2020; 08:24 GMT).

Table S8: Weather temperature (C°) in "February" countries during the study periods.

Country	Most effected cites/ capitals	March	April	May	June	July	temp1*	temp2**	temp3***	temp4***
Armenia	Yerevan	7	9	17	21	24	7	8	11	15.6
Austria	Vienna	7	12	14	19	20	7	9.5	11	14.4
Belarus	Minsk	3	6	10	19	17	3	4.5	6.3	11
Croatia	Zagreb	8	12	15	20	21	8	10	11.7	15.2
Czechia	Prague	5	10	12	17	18	5	7.5	9	12.4
Denmark	Copenhagen	5	8	11	18	16	5	6.5	8	11.6
Estonia	Tallinn	2	5	9	17	16	2	3.5	5.3	9.8
Ireland	Dublin	6	9	12	14	14	6	7.5	9	11
Lithuania	Vilnius	3	6	10	19	17	3	4.5	6.3	11
New Zealand	Wellington	16	15	13	11	10	16	15.5	14.7	13
Norway	Oslo	3	7	11	19	15	3	5	7	11
Romania	Bucharest	9	13	17	21	24	9	11	13	16.8
Switzerland	Bern	5	11	14	16	19	5	8	10	13

Source: https://www.timeanddate.com/

*the average monthly temperature during the 1st period of the study, equals to the average monthly temperature of March. ** The average monthly temperature during the 2nd period of the study, calculated as the average monthly temperature of March and April. *** The average monthly temperature during the 3rd period of the study, calculated as the average monthly temperature of March, April and May. **** The average monthly temperature during the 4th period of the study, calculated as the average monthly temperature of March, April, May and July, 2020.

Table S9: Statistics of "March" Comparable Countries.

Country	(1) Land* area (Km2)	(2) Population**	Population density (people/km2) =(2)/(1)	Median*** age	(3) Total** cases (July 8th)	(4) Total** death (July 8th)	(5) Total recovered*** (July 8th)	(6) closed cases (July 8th) =(4)+(5)	% closed cases (July 8th) =(6)/(3)	% deaths (July 8th) =(4)/(6)	Cases per million*** (July 8th)	tests per 1 million** (July 8th)
Albania	27400	2877727	105	34.3	3038	81	1744	1825	60.07%	4.44%	1056	9256
Bosnia and H.	51000	3279463	64	43.3	5621	207	2693	2900	51.59%	7.14%	1714	31313
Chile	743532	19119601	26	35.5	301019	6434	268245	274679	91.25%	2.34%	15744	63305
Jordon	88780	10204842	115	23.5	1169	10	969	979	83.75%	1.02%	115	42558
Moldova	32850	4034023	123	37.7	18141	603	11241	11844	65.29%	5.09%	4497	25083
Morocco	446300	36886364	83	29.1	14607	240	10639	10879	74.48%	2.21%	396	21705
Paraguay	397300	7133910	18	29.7	2502	20	1193	1213	48.48%	1.65%	351	11416
Peru	1280000	32979100	26	29.1	309278	10952	200938	211890	68.51%	5.17%	9378	55227
Poland	306230	37850312	124	41.9	36412	1528	24878	26406	72.52%	5.79%	962	44295
Portugal	92090	10196511	111	44.6	44416	1629	29445	31074	69.96%	5.24%	4356	124698
Saudi Arabia	2149690	34820850	16	30.8	217108	2017	154839	156856	72.25%	1.29%	6235	57972
Serbia	87460	8735110	100	43.4	16719	330	13366	13696	81.92%	2.41%	1914	52149
Slovakia	48088	5465046	114	41.8	1798	28	1473	1501	83.48%	1.87%	329	40569
Slovenia	20140	2080144	103	44.9	1739	111	1423	1534	88.21%	7.24%	836	52717
Tunisia	155360	11813725	76	32.7	1205	50	1049	1099	91.20%	4.55%	102	6227
Turkey	769630	84339554	109.58	32.2	207897	5260	185292	190552	91.66%	2.76%	2465	44257
Ukraine	579320	43724198	75	41.2	50414	1306	23119	24425	48.45%	5.35%	1153	17045
Average	427951	20914146	82	36	72534	1812	54856	56668	73.12%	3.86%	3035	41164
Minimum	20140	2080144	16	24	1169	10	969	979	48.45%	1.02%	102	6227
Maximum	2149690	84339554	124	45	309278	10952	268245	274679	91.66%	7.24%	15744	124698

* https://www.worldometers.info/geography/largest-countries-in-the-world/ (Last accessed July 8th, 2020) ** https://www.worldometers.info/coronavirus/ (Last accessed July 8th, 2020; 08:24 GMT). *** https://www.citypopulation.de/en/world/bymap/MedianAge.html (Last accessed July 8th, 2020).

Table S10: 1st case reported date, and the four time periods of the study for "March" countries

Country	1st case reported[1]	period1*	period2**	period3***	period4****
Albania	07/03/2020	25	39	69	123
Bosnia and Herzegovina	04/03/2020	28	42	72	126
Chile	02/03/2020	30	44	74	128
Jordon	01/03/2020	31	45	75	129
Moldova	06/03/2020	26	40	70	124
Morocco	01/03/2020	31	45	75	129
Paraguay	06/03/2020	26	40	70	124
Peru	05/03/2020	27	41	71	125
Poland	03/03/2020	29	43	73	127
Portugal	01/03/2020	31	45	75	129
Saudi Arabia	01/03/2020	31	45	75	129
Serbia	05/03/2020	27	41	71	125
Slovakia	05/03/2020	27	41	71	125
Slovenia	03/03/2020	29	43	73	127
Tunisia	01/03/2020	31	45	75	129
Turkey	09/03/2020	23	37	67	124
Ukraine	02/03/2020	30	44	74	128
Average		28.35	42.35	72.35	126.53
Minimum		23.00	37.00	67.00	123.00
Maximum		31.00	45.00	75.00	129.00

(1) https://www.worldometers.info/coronavirus/ (Last accessed April 1st, 2020; 00:02 GMT).

* From 1st case reported till April 1st, 2020. ** From 1st case reported till April 15th, 2020. *** From 1st case reported till May 15th, 2020. **** From 1st case reported till July 8th, 2020.

Table S11: absolute and "ln" values of the dependent variable for "March" countries, cases per million (y), at the four periods of the study.

Country	y1*	lny1	y2**	lny2	y3***	lny3	y4****	lny4
Albania	84.0	4.9	172.0	5.1	318.0	5.8	1056.0	7.0
Bosnia and H.	128.0	4.9	338.0	5.8	681.0	6.5	1714.0	7.4
Chile	143.0	5.0	414.0	6.0	2071.0	7.6	15744.0	9.7
Jordon	27.0	3.3	39.0	3.7	58.0	4.1	115.0	4.7
Moldova	88.0	4.5	479.0	6.2	1424.0	7.3	4497.0	8.4
Morocco	17.0	2.8	54.0	4.0	181.0	5.2	396.0	6.0
Paraguay	9.0	2.2	23.0	3.1	107.0	4.7	351.0	5.9
Peru	32.0	3.5	312.0	5.7	2449.0	7.8	9378.0	9.1
Poland	61.0	4.1	196.0	5.3	476.0	6.2	962.0	6.9
Portugal	730.0	6.6	1774.0	7.5	2802.0	7.9	4356.0	8.4
Saudi Arabia	45	3.8	154.0	5.0	1415.0	7.3	6235.0	8.7
Serbia	103.0	4.6	511.0	6.2	1194.0	7.1	1914.0	7.6
Slovakia	66.0	4.2	158.0	5.1	271.0	5.6	329.0	5.8
Slovenia	386.0	6.0	600.0	6.4	705.0	6.6	836.0	6.7
Tunisia	33.0	3.5	63.0	4.1	87.0	4.5	102.0	4.6
Turkey	160.0	5.1	772.0	6.6	1739.0	7.5	2465.0	7.8
Ukraine	15.0	2.7	86.0	4.5	396.0	6.0	1153.0	7.1

* https://www.worldometers.info/coronavirus/ (Last accessed April 1st, 2020; 00:02 GMT). ((https://www.worldometers.info/coronavirus/ (Last accessed April 15st, 2020; 12:36 GMT). ***https://www.worldometers.info/coronavirus/ (Last accessed May 15st, 2020; 18:08 GMT). **** https://www.worldometers.info/coronavirus/ (Last accessed July 8th, 2020; 08:24 GMT).

Table S12: Weather temperature (Cº) in "March" countries during the study periods.

Country	**Most effected cites/ capitals**	March	April	May	June	July	temp1*	temp2**	temp3***	temp4****
Albania	Tirana	12	14	19	22	25	12.0	13.0	15.0	18.4
Bosnia and H.	Sarajevo	7	14	16	20	22	7.0	10.5	12.3	15.8
Chile	Santiago	20	17	13	8	8	20.0	18.5	16.7	13.2
Jordon	Amman	13	16	23	25	26	13.0	14.5	17.3	20.6
Moldova	Chișinău	9	11	15	22	25	9.0	10.0	11.7	16.4
Morocco	Rabat	14	17	20	21	23	14.0	15.5	17.0	19.0
Paraguay	Asunción	29	24	20	21	19	29.0	26.5	24.3	22.6
Peru	Lima	24	22	19	17	16	24.0	23.0	21.7	19.6
Poland	Warsaw	5	10	12	19	20	5.0	7.5	9.0	13.2
Portugal	Lisbon	14	15	19	19	22	14.0	14.5	16.0	17.8
Saudi Arabia	Riyadh	25	30	35	38	39	25.0	27.5	30.0	33.4
Serbia	Belgrade	9	14	16	21	25	9.0	11.5	13.0	17.0
Slovakia	Bratislava	7	12	14	19	23	7.0	9.5	11.0	15.0
Slovenia	Ljubljana	7	13	15	19	22	7.0	10.0	11.7	15.2
Tunisia	Tunis	14	17	22	25	27	14.0	15.5	17.7	21.0
Turkey	Ankara	9	11	16	20	25	9.0	10.0	12.0	16.2
Ukraine	Kyiv	6	9	12	22	22	6.0	7.5	9.0	14.2

Source: https://www.timeanddate.com/

*the average monthly temperature during the 1st period of the study, equals to the average monthly temperature of March. ** The average monthly temperature during the 2nd period of the study, calculated as the average monthly temperature of March and April. *** The average monthly temperature during the 3rd period of the study, calculated as the average monthly temperature of March, April and May. **** The average monthly temperature during the 4th period of the study, calculated as the average monthly temperature of March, April, May, June and July, 2020.

Table S13: Prediction table of COVID-19 cases per million and weather temperature after 110.8 days of COVID-19 pandemic

Temp. ^{o}c	Cases per 1M	Temp. ^{o}c	Cases per 1M	Temp. ^{o}c	Cases per 1M
-5	6267	13	1263	31	254
-4	5733	14	1155	32	233
-3	5245	15	1057	33	213
-2	4798	16	967	34	195
-1	4390	17	884	35	178
0	4016	18	809	36	163
1	3674	19	740	37	149
2	3361	20	677	38	136
3	3075	21	620	39	125
4	2813	22	567	40	114
5	2573	23	519	41	104
6	2354	24	474	42	96
7	2154	25	434	43	87
8	1970	26	397	44	80
9	1803	27	363	45	73
10	1649	28	332	46	67
11	1509	29	304	47	61
12	1380	30	278	48	56

Table S14: Prediction table of COVID-19 cases per million and weather temperature after 164.8 days of COVID-19 pandemic

Temp. ^{o}c	Cases per 1M	Temp. ^{o}c	Cases per 1M	Temp. ^{o}c	Cases per 1M
-5	20414	13	2304	31	260
-4	18084	14	2041	32	230
-3	16020	15	1808	33	204
-2	14192	16	1602	34	181
-1	12572	17	1419	35	160
0	11137	18	1257	36	142
1	9866	19	1113	37	126
2	8739	20	986	38	111
3	7742	21	874	39	99
4	6858	22	774	40	87
5	6075	23	686	41	77
6	5382	24	607	42	69
7	4768	25	538	43	61
8	4223	26	477	44	54
9	3741	27	422	45	48
10	3314	28	374	46	42
11	2936	29	331	47	37
12	2601	30	294	48	33

Table S15: Statistics of "March" Comparable Countries –in case of including Togo-.

Country	(1) Land* area (Km2)	(2) Population**	Population density (people/km2) =(2)/(1)	Median*** age	(3) Total** cases (July 8th)	(4) Total** death (July 8th)	(5) Total recovered*** (July 8th)	(6) closed cases (July 8th) =(4)+(5)	% closed cases (July 8th) =(6)/(3)	% deaths (July 8th) =(4)/(6)	Cases per million*** (July 8th)	tests per 1 million** (July 8th)
Albania	27400	2877727	105	34.3	3038	81	1744	1825	60.07%	4.44%	1056	9256
Bosnia and H.	51000	3279463	64	43.3	5621	207	2693	2900	51.59%	7.14%	1714	31313
Chile	743532	19119601	26	35.5	301019	6434	268245	274679	91.25%	2.34%	15744	63305
Jordon	88780	10204842	115	23.5	1169	10	969	979	83.75%	1.02%	115	42558
Moldova	32850	4034023	123	37.7	18141	603	11241	11844	65.29%	5.09%	4497	25083
Morocco	446300	36886364	83	29.1	14607	240	10639	10879	74.48%	2.21%	396	21705
Paraguay	397300	7133910	18	29.7	2502	20	1193	1213	48.48%	1.65%	351	11416
Peru	1280000	32979100	26	29.1	309278	10952	200938	211890	68.51%	5.17%	9378	55227
Poland	306230	37850312	124	41.9	36412	1528	24878	26406	72.52%	5.79%	962	44295
Portugal	92090	10196511	111	44.6	44416	1629	29445	31074	69.96%	5.24%	4356	124698
Saudi Arabia	2149690	34820850	16	30.8	217108	2017	154839	156856	72.25%	1.29%	6235	57972
Serbia	87460	8735110	100	43.4	16719	330	13366	13696	81.92%	2.41%	1914	52149
Slovakia	48088	5465046	114	41.8	1798	28	1473	1501	83.48%	1.87%	329	40569
Slovenia	20140	2080144	103	44.9	1739	111	1423	1534	88.21%	7.24%	836	52717
South Africa	1213090	59317120	49	28	215855	3502	102299	105801	49.01%	3.31%	3639	32156
Togo	54390	8301205	153	20	689	15	467	482	69.96%	3.11%	83	4025
Tunisia	155360	11813725	76	32.7	1205	50	1049	1099	91.20%	4.55%	102	6227
Ukraine	579320	43724198	75	41.2	50414	1306	23119	24425	48.45%	5.35%	1153	17045
Average	431834.4	18823292	82.20	35.08	68985	1615	47223	48838	70.6%	3.8%	3937	38428.67
Minimum	20140	2080143.541	16.20	20	689	10	467	482	48.4%	1.0%	83	4025
Maximum	2149690	59317120.09	152.62	44.9	309278	10952	268245	274679	91.2%	7.2%	15744	124698

* https://www.worldometers.info/geography/largest-countries-in-the-world/ (Last accessed July 8th, 2020) ** https://www.worldometers.info/coronavirus/ (Last accessed July 8th, 2020; 08:24 GMT). *** https://www.citypopulation.de/en/world/bymap/MedianAge.html (Last accessed July 8th, 2020).

Table S16: 1st case reported date, and the four time periods of the study for "March" countries –in case of including togo-

Country	1st case reported[1]	period1*	period2**	period3***	period4****
Albania	07/03/2020	25	39	69	123
Bosnia and H.	04/03/2020	28	42	72	126
Chile	02/03/2020	30	44	74	128
Jordon	01/03/2020	31	45	75	129
Moldova	06/03/2020	26	40	70	124
Morocco	01/03/2020	31	45	75	129
Paraguay	06/03/2020	26	40	70	124
Peru	05/03/2020	27	41	71	125
Poland	03/03/2020	29	43	73	127
Portugal	01/03/2020	31	45	75	129
Saudi Arabia	01/03/2020	31	45	75	129
Serbia	05/03/2020	27	41	71	125
Slovakia	05/03/2020	27	41	71	125
Slovenia	03/03/2020	29	43	73	127
South Africa	04/03/2020	28	42	72	126
Togo	05/03/2020	27	41	71	125
Tunisia	01/03/2020	31	45	75	129
Ukraine	02/03/2020	30	44	74	128
**Average**		**28.56**	**42.56**	**72.56**	**126.56**
Minimum		25.00	39.00	69.00	123.00
Maximum		31.00	45.00	75.00	129.00

(1) https://www.worldometers.info/coronavirus/ (Last accessed April 1st, 2020; 00:02 GMT).

* From 1st case reported till April 1st, 2020. ** From 1st case reported till April 15th, 2020. *** From 1st case reported till May 15th, 2020. **** From 1st case reported till July 8th, 2020.

Table S17: absolute and "ln" values of the dependent variable for "March" countries, cases per million (y), at the four periods of the study –in case of including Togo-.

Country	y1*	lny1	y2**	lny2	y3***	lny3	y4****	lny4
Albania	84	3.5	172	5.15	318	5.76	1056	6.96
Bosnia and H.	128	4.85	338	5.82	681	6.52	1714	7.45
Chile	143	4.96	414	6.03	2071	7.64	15744	9.66
Jordon	27	3.3	39	3.66	58	4.06	115	4.74
Moldova	88	4.48	479	6.17	1424	7.26	4497	8.41
Morocco	17	2.83	54	3.99	181	5.2	396	5.98
Paraguay	9	2.2	23	3.14	107	4.67	351	5.86
Peru	32	3.47	312	5.74	2449	7.8	9378	9.15
Poland	61	4.11	196	5.28	476	6.17	962	6.87
Portugal	730	6.59	1774	7.48	2802	7.94	4356	8.38
Saudi Arabia	143	4.96	154	5.04	1415	7.25	6235	8.74
Serbia	103	4.63	511	6.24	1194	7.09	1914	7.56
Slovakia	66	4.19	158	5.06	271	5.6	329	5.8
Slovenia	386	5.96	600	6.4	705	6.56	836	6.73
South Africa	23	3.14	41	3.71	215	5.37	3639	8.2
Togo	4	1.39	10	2.3	29	3.37	83	4.42
Tunisia	33	1.39	63	4.14	87	4.47	102	4.62
Ukraine	15	2.71	86	4.45	396	5.98	1153	7.05

* https://www.worldometers.info/coronavirus/ (Last accessed April 1st, 2020; 00:02 GMT). ((https://www.worldometers.info/coronavirus/ (Last accessed April 15st, 2020; 12:36 GMT). ***https://www.worldometers.info/coronavirus/ (Last accessed May 15st, 2020; 18:08 GMT). **** https://www.worldometers.info/coronavirus/ (Last accessed July 8th, 2020; 08:24 GMT).

Table S18: Weather temperature (C°) in "March" countries during the study periods –in case of including Togo-.

Country	Most effected cites/ capitals	March	April	May	June	July	temp1*	temp2**	temp3***	temp4****
Albania	Tirana	12	14	19	22	26	12	13	15	18.6
Bosnia and H.	Sarajevo	7	14	16	20	22	7	10.5	12.3	15.8
Chile	Santiago	20	17	13	8	8	20	18.5	16.7	13.2
Jordon	Amman	13	16	23	25	26	13	14.5	17.3	20.6
Moldova	Chișinău	9	11	15	22	25	9	10	11.7	16.4
Morocco	Rabat	14	17	20	21	23	14	15.5	17	19
Paraguay	Asunción	29	24	20	21	16	29	26.5	24.3	22
Peru	Lima	24	22	19	17	16	24	23	21.7	19.6
Poland	Warsaw	5	10	12	19	20	5	14.5	16	17.8
Portugal	Lisbon	14	15	19	19	22	14	14.5	16	17.8
Saudi Arabia	Riyadh	25	30	35	38	39	25	27.5	30	33.4
Serbia	Belgrade	9	14	16	21	25	9	11.5	13	17
Slovakia	Bratislava	7	12	14	19	23	7	7.5	9	13.2
Slovenia	Ljubljana	7	13	15	19	22	7	10	11.7	15.2
South Africa	Cape town	20	17	15	14	13	20	18.5	17.3	15.8
Togo	Lome	29	29	28	27	26	29	29	28.7	27.8
Tunisia	Tunis	14	17	22	25	27	14	15.5	17.7	21
Ukraine	Kyiv	6	9	12	22	22	6	7.5	9	14.2

Source: https://www.timeanddate.com/

*the average monthly temperature during the 1st period of the study, equals to the average monthly temperature of March. ** The average monthly temperature during the 2nd period of the study, calculated as the average monthly temperature of March and April. *** The average monthly temperature during the 3rd period of the study, calculated as the average monthly temperature of March, April and May. **** The average monthly temperature during the 4th period of the study, calculated as the average monthly temperature of March, April, May, June and July, 2020.

Table 19: Results of the regression models for "March" countries including Togo and South Africa instead of Turkey

Days [(1)]	Actual No. of observations [(2)]	R^2_{adj}	F	Pro. > F	Intercept (S.E.)	P-value	Temp.(X) (S.E.)	P-value
28.56	13	0.338	7.12	0.0219	5.125 (0.490)	0.000	-0.0852 (0.032)	0.022**
42.56	13	0.386	8.54	0.0139	7.146 (0.780)	0.000	-0.0151 (0.052)	0.014*
72.56	15	0.101	258	0.1325	7.810 (1.151)	0.000	-0.1200 (0.075)	0.133
126.56	15	0.044	1.64	0.2228	9.990 (2.423)	0.001	-0.1746 (0.136)	0.223

(1) Days from the 1st case reported. (2) After excluding outliers, leverage and influencer observations.

* Significant at 1% significance level. ** Significant at 5% significance level.

By substituting the regression parameters for March countries "after 28.56 days and 42.56" in equation 3, we obtained equations 4 and 5:

$$lny_{it} = 5.125 - 0.0852\, x_{it} \ldots\ldots\ldots\ldots (8)$$

$$lny_{it} = 7.146 - 0.0151\, x_{it} \ldots\ldots\ldots\ldots (9)$$

Applying e on both sides:

$$y_{it} = (e^{-0.0852})^{x_{it}} + e^{5.125} = EXP\ (-0.0852)^{x_{it}} + EXP\ (5.125) \ldots\ldots\ldots\ldots (10)$$

$$y_{it} = (e^{-0.0151})^{x_{it}} + e^{7.146} = EXP\ (-0.0151)^{x_{it}} + EXP\ (7.146) \ldots\ldots\ldots\ldots (11)$$

Equations 6 and 7 used for predicting the development of COVID-19 cases per million in terms of weather temperature (Tables S20 and S21).

Table S20: Prediction table of COVID-19 cases per million and weather temperature after 28.56 days of COVID-19 pandemic

Temp. ^{o}c	Cases per 1M	Temp. ^{o}c	Cases per 1M	Temp. ^{o}c	Cases per 1M
-5	257	13	56	31	12
-4	236	14	51	32	11
-3	217	15	47	33	10
-2	199	16	43	34	9
-1	183	17	40	35	9
0	168	18	36	36	8
1	154	19	33	37	7
2	142	20	31	38	7
3	130	21	28	39	6
4	120	22	26	40	6
5	110	23	24	41	5
6	101	24	22	42	5
7	93	25	20	43	4
8	85	26	18	44	4
9	78	27	17	45	4
10	72	28	15	46	3
11	66	29	14	47	3
12	60	30	13	48	3

Table S21: Prediction table of COVID-19 cases per million and weather temperature after 42.56 days of COVID-19 pandemic

Temp. ^{o}c	Cases per 1M	Temp. ^{o}c	Cases per 1M	Temp. ^{o}c	Cases per 1M
-5	1369	13	1043	31	795
-4	1348	14	1027	32	783
-3	1328	15	1012	33	771
-2	1308	16	997	34	759
-1	1288	17	982	35	748
0	1269	18	967	36	737
1	1250	19	953	37	726
2	1231	20	938	38	715
3	1213	21	924	39	704
4	1195	22	910	40	694
5	1177	23	897	41	683
6	1159	24	883	42	673
7	1142	25	870	43	663
8	1125	26	857	44	653
9	1108	27	844	45	643
10	1091	28	831	46	634
11	1075	29	819	47	624
12	1059	30	807	48	615

Table S22: Observed and estimated COVID-19 cases per million in terms of temperature in "March-group" countries –in case of including Togo and South Africa instead of Turkey-

Countries	After 28.56 days in average			After 42.56 days in average		
	Temp. (C°)	COVID−19 cases per 1M		Temp. (C°)	COVID−19 cases per 1M	
		Observed	Estimated in terms of weather temp.		Observed	Estimated in terms of weather temp.
Albania	12	84	60	13	172	1043
Bosnia and H.	7	128	93	10.5	338	1083
Chile	20	143	31	18.5	414	960
Jordon	13	27	56	14.5	39	1019
Moldova	9	88	78	10	479	1091
Morocco	14	17	51	15.5	54	1004
Paraguay	29	9	14	26.5	23	851
Peru	24	32	22	23	312	897
Poland	5	61	110	14.5	196	1019
Portugal	14	730	51	14.5	1774	1019
Saudi Arabia	25	143	20	27.5	154	838
Serbia	9	103	78	11.5	511	1067
Slovakia	7	66	93	7.5	158	1133
Slovenia	7	386	93	10	600	1091
South Africa	20	23	31	18.5	41	960
Togo	29	4	14	29	10	819
Tunisia	14	33	51	15.5	63	1004
Ukraine	6	15	101	7.5	86	1133

Observed cases / million is less than estimated (green)
Observed cases / million is higher than estimated (red)
