# Supplementary material for: Does Temperature Affect COVID-19 Transmission?
Source: Front Public Health. 2020 Dec 22;8:554964. doi: 10.3389/fpubh.2020.554964 (PMC7793668; doi:10.3389/fpubh.2020.554964)
Supplement: Supplementary file 2 [file Data_Sheet_2.PDF]

```
                                                     ___  ____  ____  ____  ____(R)
                                                    /__    /   ____/   /   ____/
                                                   ___/   /   /___/   /   /___/
                                                     Statistics/Data analysis
```

Project: Does Weather Temperature affect COVID-19 Transmission?

```
  ___  ____  ____  ____  ____ (R)
 /__    /   ____/   /   ____/
___/   /   /___/   /   /___/   16.1   Copyright 1985-2019 StataCorp LLC
  Statistics/Data analysis            StataCorp
                                      4905 Lakeway Drive
     MP - Parallel Edition            College Station, Texas 77845 USA
                                      800-STATA-PC        https://www.stata.com
                                      979-696-4600        stata@stata.com
                                      979-696-4601 (fax)

Stata license: Single-user 2-core network, expiring 17 Sep 2020
Serial number: 501609327014
  Licensed to:

Notes:
      1. Unicode is supported; see help unicode_advice.
      2. More than 2 billion observations are allowed; see help obs_advice.
      3. Maximum number of variables is set to 5,000; see help set_maxvar.
      4. New update available; type -update all-

  1 . import excel "E:\انتا ج علمى\IN PROGRESS\كورونا \Analysis\results\STATA 16.1\B
  > ook1.xlsx", sheet("February") firstrow
  (9 vars, 13 obs)

  2 . do "C:\Users\HP\AppData\Local\Temp\STD2598_000000.tmp"

  3 . reg lny1 temp1
```

Source	SS	df	MS			
				Number of obs	=	13
				F(1, 11)	=	0.29
Model	.473347978	1	.473347978	Prob > F	=	0.5992
Residual	17.7772828	11	1.61611662	R-squared	=	0.0259
				Adj R-squared	=	-0.0626
Total	18.2506308	12	1.5208859	Root MSE	=	1.2713

lny1	Coef.	Std. Err.	t	P>|t|	[95% Conf.	Interval]
temp1	-.0542352	.1002137	-0.54	0.599	-.2748041	.1663337
_cons	6.047275	.7036951	8.59	0.000	4.498453	7.596098

```
  4 . predict d, cooksd

  5 . sort d

  6 . br country d

  7 .
  end of do-file

  8 . do "C:\Users\HP\AppData\Local\Temp\STD2598_000000.tmp"

  9 . predict diffits, dfits

 10 . generate absdiffits=abs(diffits)

 11 . sort absdiffits
```

```
12 . br country absdiffits

13 .
   end of do-file

14 . do "C:\Users\HP\AppData\Local\Temp\STD2598_000000.tmp"

15 . drop in 12/13
   (2 observations deleted)

16 . reg lny1 temp1
```

Source	SS	df	MS			
				Number of obs	=	11
				F(1, 9)	=	1.53
Model	1.10565597	1	1.10565597	Prob > F	=	0.2472
Residual	6.49854403	9	.722060448	R-squared	=	0.1454
				Adj R-squared	=	0.0504
Total	7.6042	10	.76042	Root MSE	=	.84974

lny1	Coef.	Std. Err.	t	P>|t|	[95% Conf.	Interval]
temp1	-.1506343	.1217309	-1.24	0.247	-.4260087	.12474
_cons	6.881642	.7117022	9.67	0.000	5.27166	8.491624

```
17 .
   end of do-file

18 . import excel "E:\انتاج علمى\IN PROGRESS\كورونا \Analysis\results\STATA 16.1\B
   > ook1.xlsx", sheet("February") firstrow clear
   (9 vars, 13 obs)

19 . do "C:\Users\HP\AppData\Local\Temp\STD2598_000000.tmp"

20 . reg lny2 temp2
```

Source	SS	df	MS			
				Number of obs	=	13
				F(1, 11)	=	1.01
Model	.625262386	1	.625262386	Prob > F	=	0.3362
Residual	6.80196838	11	.618360762	R-squared	=	0.0842
				Adj R-squared	=	0.0009
Total	7.42723077	12	.618935897	Root MSE	=	.78636

lny2	Coef.	Std. Err.	t	P>|t|	[95% Conf.	Interval]
temp2	-.0700816	.0696937	-1.01	0.336	-.2234764	.0833133
_cons	7.172172	.5837399	12.29	0.000	5.887369	8.456975

```
21 . predict d, cooksd

22 . sort d

23 . br country d

24 .
   end of do-file
```

```
25 . do "C:\Users\HP\AppData\Local\Temp\STD2598_000000.tmp"

26 . predict diffits, dfits

27 . generate absdiffits=abs(diffits)

28 . sort absdiffits

29 . br country absdiffits

30 .
   end of do-file

31 . do "C:\Users\HP\AppData\Local\Temp\STD2598_000000.tmp"

32 . drop in 12/13
   (2 observations deleted)

33 . reg lny2 temp2

      Source |       SS           df       MS      Number of obs   =        11
-------------+----------------------------------   F(1, 9)         =      0.14
       Model |  .069386159         1  .069386159   Prob > F        =    0.7171
    Residual |  4.46650475         9  .496278306   R-squared       =    0.0153
-------------+----------------------------------   Adj R-squared   =   -0.0941
       Total |  4.53589091        10  .453589091   Root MSE        =    .70447

------------------------------------------------------------------------------
        lny2 |      Coef.   Std. Err.      t    P>|t|     [95% Conf. Interval]
-------------+----------------------------------------------------------------
       temp2 |  -.0335272   .0896652    -0.37   0.717    -.2363641    .1693096
       _cons |   6.825306   .6664848    10.24   0.000     5.317612    8.332999
------------------------------------------------------------------------------

34 .
   end of do-file

35 . import excel "E:\انتا ج علمى\IN PROGRESS\كورونا \Analysis\results\STATA 16.1\B
   > ook1.xlsx", sheet("February") firstrow clear
   (9 vars, 13 obs)

36 . do "C:\Users\HP\AppData\Local\Temp\STD2598_000000.tmp"

37 . reg lny3 temp3

      Source |       SS           df       MS      Number of obs   =        13
-------------+----------------------------------   F(1, 11)        =      2.23
       Model |  1.28483106         1  1.28483106   Prob > F        =    0.1633
    Residual |  6.33227664        11  .575661512   R-squared       =    0.1687
-------------+----------------------------------   Adj R-squared   =    0.0931
       Total |  7.61710769        12  .634758974   Root MSE        =    .75872

------------------------------------------------------------------------------
        lny3 |      Coef.   Std. Err.      t    P>|t|     [95% Conf. Interval]
-------------+----------------------------------------------------------------
       temp3 |  -.1157546   .0774816    -1.49   0.163    -.2862904    .0547813
       _cons |   8.255137   .7586901    10.88   0.000     6.585271    9.925003
------------------------------------------------------------------------------

38 . predict d, cooksd
```

```
39 . sort d

40 . br country d

41 .
   end of do-file

42 . do "C:\Users\HP\AppData\Local\Temp\STD2598_000000.tmp"

43 . predict diffits, dfits

44 . generate absdiffits=abs(diffits)

45 . sort absdiffits

46 . br country absdiffits

47 .
   end of do-file

48 . do "C:\Users\HP\AppData\Local\Temp\STD2598_000000.tmp"

49 . drop in 11/13
   (3 observations deleted)

50 . reg lny3 temp3

         Source |       SS           df       MS      Number of obs   =        10
   -------------+----------------------------------   F(1, 8)         =      1.62
          Model |  .513277151         1  .513277151   Prob > F        =    0.2384
       Residual |  2.53012285         8  .316265356   R-squared       =    0.1687
   -------------+----------------------------------   Adj R-squared   =    0.0647
          Total |      3.0434         9  .338155556   Root MSE        =    .56237

   ------------------------------------------------------------------------------
           lny3 |      Coef.   Std. Err.      t    P>|t|     [95% Conf. Interval]
   -------------+----------------------------------------------------------------
          temp3 |  -.0942831   .0740089    -1.27   0.238    -.2649478    .0763816
          _cons |   8.130233   .7058714    11.52   0.000     6.502491    9.757975
   ------------------------------------------------------------------------------

51 .
   end of do-file

52 . import excel "E:\ا نتا ج علمى\IN PROGRESS\انوروك \Analysis\results\STATA 16.1\B
   > ook1.xlsx", sheet("February") firstrow clear
   (9 vars, 13 obs)

53 . do "C:\Users\HP\AppData\Local\Temp\STD2598_000000.tmp"

54 . reg lny4 temp4

         Source |       SS           df       MS      Number of obs   =        13
   -------------+----------------------------------   F(1, 11)        =      0.00
          Model |  .002093398         1  .002093398   Prob > F        =    0.9649
       Residual |  11.3598143        11  1.03271039   R-squared       =    0.0002
   -------------+----------------------------------   Adj R-squared   =   -0.0907
          Total |  11.3619077        12  .946825641   Root MSE        =    1.0162

   ------------------------------------------------------------------------------
           lny4 |      Coef.   Std. Err.      t    P>|t|     [95% Conf. Interval]
   -------------+----------------------------------------------------------------
          temp4 |   .0061266   .1360755     0.05   0.965    -.2933736    .3056267
          _cons |   7.478017   1.758224     4.25   0.001     3.608192    11.34784
   ------------------------------------------------------------------------------
```

```
55 . predict d, cooksd

56 . sort d

57 . br country d

58 .
   end of do-file

59 . do "C:\Users\HP\AppData\Local\Temp\STD2598_000000.tmp"

60 . predict diffits, dfits

61 . generate absdiffits=abs(diffits)

62 . sort absdiffits

63 . br country absdiffits

64 .
   end of do-file

65 . do "C:\Users\HP\AppData\Local\Temp\STD2598_000000.tmp"

66 . drop in 12/13
   (2 observations deleted)

67 . reg lny4 temp4
```

Source	SS	df	MS
Model	**.325192193**	**1**	**.325192193**
Residual	**4.93109872**	**9**	**.547899857**
Total	**5.25629091**	**10**	**.525629091**

Number of obs	=	**11**
F(1, 9)	=	**0.59**
Prob > F	=	**0.4608**
R-squared	=	**0.0619**
Adj R-squared	=	**-0.0424**
Root MSE	=	**.7402**

lny4	Coef.	Std. Err.	t	P>|t|	[95% Conf.	Interval]
temp4	**-.0834098**	**.1082674**	**-0.77**	**0.461**	**-.3283278**	**.1615081**
_cons	**8.611257**	**1.368708**	**6.29**	**0.000**	**5.515024**	**11.70749**

```
68 .
   end of do-file

69 .
```
