# Supplementary material for: Does Temperature Affect COVID-19 Transmission?
Source: Front Public Health. 2020 Dec 22;8:554964. doi: 10.3389/fpubh.2020.554964 (PMC7793668; doi:10.3389/fpubh.2020.554964)
Supplement: Supplementary file 3 [file Data_Sheet_3.PDF]

```
                                                                       ___  ____  ____  ____  ____(R)
                                                                      /__    /   ____/   /   ____/
                                                                     ___/   /   /___/   /   /___/
                                                                       Statistics/Data analysis
```

Project: Does Weather Temperature affect COVID-19 Transmission?

```
  ___  ____  ____  ____  ____ (R)
 /__    /   ____/   /   ____/
___/   /   /___/   /   /___/   16.1   Copyright 1985-2019 StataCorp LLC
  Statistics/Data analysis            StataCorp
                                      4905 Lakeway Drive
     MP - Parallel Edition            College Station, Texas 77845 USA
                                      800-STATA-PC        https://www.stata.com
                                      979-696-4600        stata@stata.com
                                      979-696-4601 (fax)

Stata license: Single-user 2-core network, expiring 17 Sep 2020
Serial number: 501609327014
  Licensed to:


Notes:
      1. Unicode is supported; see help unicode_advice.
      2. More than 2 billion observations are allowed; see help obs_advice.
      3. Maximum number of variables is set to 5,000; see help set_maxvar.
      4. New update available; type -update all-

  1 . import excel "E:\ا نتا ج علمی\IN PROGRESS\انورونک\Analysis\results\STATA 16.1\B
    > ook1.xlsx", sheet("January") firstrow
    (9 vars, 13 obs)

  2 . do "C:\Users\HP\AppData\Local\Temp\STD3cc4_000000.tmp"

  3 . reg lny1 temp1
```

Source	SS	df	MS			
				Number of obs	=	13
				F(1, 11)	=	0.06
Model	.147171578	1	.147171578	Prob > F	=	0.8170
Residual	28.8021207	11	2.61837461	R-squared	=	0.0051
				Adj R-squared	=	-0.0854
Total	28.9492923	12	2.41244103	Root MSE	=	1.6181

lny1	Coef.	Std. Err.	t	P>|t|	[95% Conf.	Interval]
temp1	-.0136206	.0574512	-0.24	0.817	-.1400699	.1128287
_cons	5.725115	.6902888	8.29	0.000	4.205799	7.24443

```
  4 . predict d, cooksd

  5 . sort d

  6 . br country d

  7 .
    end of do-file

  8 . do "C:\Users\HP\AppData\Local\Temp\STD3cc4_000000.tmp"

  9 . predict diffits, dfits

 10 . generate absdiffits=abs(diffits)

 11 . sort absdiffits
```

```
12 . br country absdiffits

13 . drop in 11/13
   (3 observations deleted)

14 .
   end of do-file

15 . do "C:\Users\HP\AppData\Local\Temp\STD3cc4_000000.tmp"

16 . reg lny1 temp1

      Source |       SS           df       MS      Number of obs   =        10
-------------+----------------------------------   F(1, 8)         =      0.04
       Model |  .030693184         1  .030693184   Prob > F        =    0.8503
    Residual |  6.46479682         8  .808099602   R-squared       =    0.0047
-------------+----------------------------------   Adj R-squared   =   -0.1197
       Total |     6.49549         9  .721721111   Root MSE        =    .89894

------------------------------------------------------------------------------
        lny1 |      Coef.   Std. Err.      t    P>|t|     [95% Conf. Interval]
-------------+----------------------------------------------------------------
       temp1 |  -.0097342   .0499474    -0.19   0.850    -.1249131    .1054447
       _cons |   6.366562   .4890795    13.02   0.000     5.238743    7.494382
------------------------------------------------------------------------------

17 .
   end of do-file

18 . import excel "E:\انتاج علمى\IN PROGRESS\كورونا \Analysis\results\STATA 16.1\B
   > ook1.xlsx", sheet("January") firstrow clear
   (9 vars, 13 obs)

19 . do "C:\Users\HP\AppData\Local\Temp\STD3cc4_000000.tmp"

20 . reg lny2 temp2

      Source |       SS           df       MS      Number of obs   =        13
-------------+----------------------------------   F(1, 11)        =      0.07
       Model |  .119176438         1  .119176438   Prob > F        =    0.7925
    Residual |  18.0416543        11  1.64015039   R-squared       =    0.0066
-------------+----------------------------------   Adj R-squared   =   -0.0838
       Total |  18.1608308        12  1.51340256   Root MSE        =    1.2807

------------------------------------------------------------------------------
        lny2 |      Coef.   Std. Err.      t    P>|t|     [95% Conf. Interval]
-------------+----------------------------------------------------------------
       temp2 |  -.0130283    .048332    -0.27   0.792    -.1194063    .0933497
       _cons |   6.702591   .5998029    11.17   0.000     5.382434    8.022748
------------------------------------------------------------------------------

21 . predict d, cooksd

22 . sort d

23 . br country d

24 .
   end of do-file
```

```
25 . do "C:\Users\HP\AppData\Local\Temp\STD3cc4_000000.tmp"

26 . predict diffits, dfits

27 . generate absdiffits=abs(diffits)

28 . sort absdiffits

29 . br country absdiffits

30 .
   end of do-file

31 . do "C:\Users\HP\AppData\Local\Temp\STD3cc4_000000.tmp"

32 . drop in 12/13
   (2 observations deleted)

33 .
   end of do-file

34 . do "C:\Users\HP\AppData\Local\Temp\STD3cc4_000000.tmp"

35 . reg lny2 temp2

         Source |       SS           df       MS      Number of obs   =        11
   -------------+----------------------------------   F(1, 9)         =      1.01
          Model |  .89161618         1  .89161618   Prob > F        =    0.3422
       Residual |  7.98303837         9  .887004263   R-squared       =    0.1005
   -------------+----------------------------------   Adj R-squared   =    0.0005
          Total |  8.87465455        10  .887465455   Root MSE        =    .94181

   ------------------------------------------------------------------------------
           lny2 |      Coef.   Std. Err.      t    P>|t|     [95% Conf. Interval]
   -------------+----------------------------------------------------------------
          temp2 |  -.0374368   .0373398    -1.00   0.342    -.1219053    .0470318
          _cons |   7.325231   .4909996    14.92   0.000     6.214513    8.435949
   ------------------------------------------------------------------------------

36 .
   end of do-file

37 . import excel "E:\انتاج علمى\IN PROGRESS\كورونا \Analysis\results\STATA 16.1\B
   > ook1.xlsx", sheet("January") firstrow clear
   (9 vars, 13 obs)

38 . do "C:\Users\HP\AppData\Local\Temp\STD3cc4_000000.tmp"

39 . reg lny3 temp3

         Source |       SS           df       MS      Number of obs   =        13
   -------------+----------------------------------   F(1, 11)        =      3.22
          Model |  4.85212002         1  4.85212002   Prob > F        =    0.1004
       Residual |  16.5907569        11  1.50825063   R-squared       =    0.2263
   -------------+----------------------------------   Adj R-squared   =    0.1559
          Total |  21.4428769        12  1.78690641   Root MSE        =    1.2281

   ------------------------------------------------------------------------------
           lny3 |      Coef.   Std. Err.      t    P>|t|     [95% Conf. Interval]
   -------------+----------------------------------------------------------------
          temp3 |  -.0906216   .0505246    -1.79   0.100    -.2018256    .0205823
          _cons |   8.129434   .6611466    12.30   0.000      6.67426    9.584607
   ------------------------------------------------------------------------------
```

```
40 . predict d, cooksd

41 . sort d

42 . br country d

43 .
   end of do-file

44 . do "C:\Users\HP\AppData\Local\Temp\STD3cc4_000000.tmp"

45 . predict diffits, dfits

46 . generate absdiffits=abs(diffits)

47 . sort absdiffits

48 . br country absdiffits

49 .
   end of do-file

50 . drop in 13
   (1 observation deleted)

51 . do "C:\Users\HP\AppData\Local\Temp\STD3cc4_000000.tmp"

52 . reg lny3 temp3
```

Source	SS	df	MS			
				Number of obs	=	**12**
				F(1, 10)	=	**4.20**
Model	**4.6832502**	**1**	**4.6832502**	Prob > F	=	**0.0677**
Residual	**11.1621748**	**10**	**1.11621748**	R-squared	=	**0.2956**
				Adj R-squared	=	**0.2251**
Total	**15.845425**	**11**	**1.44049318**	Root MSE	=	**1.0565**

lny3	Coef.	Std. Err.	t	P>|t|	[95% Conf.	Interval]
temp3	**-.0890428**	**.043471**	**-2.05**	**0.068**	**-.1859022**	**.0078166**
_cons	**8.298295**	**.5738994**	**14.46**	**0.000**	**7.019568**	**9.577023**

```
53 .
   end of do-file

54 . import excel "E:\ا نتا ج علمى\IN PROGRESS\انوروك \Analysis\results\STATA 16.1\B
   > ook1.xlsx", sheet("January") firstrow clear
   (9 vars, 13 obs)

55 . do "C:\Users\HP\AppData\Local\Temp\STD3cc4_000000.tmp"

56 . reg lny4 temp4
```

Source	SS	df	MS			
				Number of obs	=	**13**
				F(1, 11)	=	**3.82**
Model	**6.36121764**	**1**	**6.36121764**	Prob > F	=	**0.0766**
Residual	**18.3217516**	**11**	**1.66561378**	R-squared	=	**0.2577**
				Adj R-squared	=	**0.1902**
Total	**24.6829692**	**12**	**2.0569141**	Root MSE	=	**1.2906**

lny4	Coef.	Std. Err.	t	P>|t|	[95% Conf.	Interval]
temp4	**-.1286161**	**.0658131**	**-1.95**	**0.077**	**-.2734698**	**.0162376**
_cons	**9.230784**	**.9797452**	**9.42**	**0.000**	**7.074379**	**11.38719**

```
57 . predict d, cooksd

58 . sort d

59 . br country d

60 .
   end of do-file

61 . do "C:\Users\HP\AppData\Local\Temp\STD3cc4_000000.tmp"

62 . predict diffits, dfits

63 . generate absdiffits=abs(diffits)

64 . sort absdiffits

65 . br country absdiffits

66 .
   end of do-file

67 . do "C:\Users\HP\AppData\Local\Temp\STD3cc4_000000.tmp"

68 . drop in 13
   (1 observation deleted)

69 .
   end of do-file

70 . do "C:\Users\HP\AppData\Local\Temp\STD3cc4_000000.tmp"

71 . reg lny4 temp4

         Source |       SS           df       MS      Number of obs   =        12
   -------------+----------------------------------   F(1, 10)        =      4.39
          Model |  5.64437816         1  5.64437816   Prob > F        =    0.0626
       Residual |  12.8584468        10  1.28584468   R-squared       =    0.3051
   -------------+----------------------------------   Adj R-squared   =    0.2356
          Total |   18.502825        11    1.682075   Root MSE        =     1.134

   ------------------------------------------------------------------------------
           lny4 |      Coef.   Std. Err.      t    P>|t|     [95% Conf. Interval]
   -------------+----------------------------------------------------------------
          temp4 |  -.1213761   .0579321    -2.10   0.063    -.2504568    .0077047
          _cons |   9.317938   .8618734    10.81   0.000     7.397564    11.23831
   ------------------------------------------------------------------------------

72 .
   end of do-file

73 .
```
