# Supplementary material for: Does Temperature Affect COVID-19 Transmission?
Source: Front Public Health. 2020 Dec 22;8:554964. doi: 10.3389/fpubh.2020.554964 (PMC7793668; doi:10.3389/fpubh.2020.554964)
Supplement: Supplementary file 4 [file Data_Sheet_4.PDF]

```
                                                     ___  ____  ____  ____  ____(R)
                                                    /__    /   ____/   /   ____/
                                                   ___/   /   /___/   /   /___/
                                                     Statistics/Data analysis

                                Project: Does Weather Temperature affect COVID-19 Transmission?

  ___  ____  ____  ____  ____ (R)
 /__    /   ____/   /   ____/
___/   /   /___/   /   /___/   16.1   Copyright 1985-2019 StataCorp LLC
  Statistics/Data analysis            StataCorp
                                      4905 Lakeway Drive
     MP - Parallel Edition            College Station, Texas 77845 USA
                                      800-STATA-PC        https://www.stata.com
                                      979-696-4600        stata@stata.com
                                      979-696-4601 (fax)

Stata license: Single-user 2-core network, expiring 17 Sep 2020
Serial number: 501609327014
  Licensed to:


Notes:
      1. Unicode is supported; see help unicode_advice.
      2. More than 2 billion observations are allowed; see help obs_advice.
      3. Maximum number of variables is set to 5,000; see help set_maxvar.
      4. New update available; type -update all-

  1 . import excel "E:\ا نتا ج علمی\IN PROGRESS\كورونا \Analysis\results\STATA 16.1\B
> ook1.xlsx", sheet("March") firstrow
(9 vars, 17 obs)

  2 . do "C:\Users\HP\AppData\Local\Temp\STD31f4_000000.tmp"

  3 . reg lny1 temp1

      Source |       SS           df       MS      Number of obs   =        17
-------------+----------------------------------   F(1, 15)        =      2.30
       Model |  2.90288281         1  2.90288281   Prob > F        =    0.1503
    Residual |  18.9418231        15   1.2627882   R-squared       =    0.1329
-------------+----------------------------------   Adj R-squared   =    0.0751
       Total |  21.8447059        16  1.36529412   Root MSE        =    1.1237

------------------------------------------------------------------------------
        lny1 |      Coef.   Std. Err.      t    P>|t|     [95% Conf. Interval]
-------------+----------------------------------------------------------------
       temp1 |  -.0586999   .0387158    -1.52   0.150    -.1412206    .0238208
       _cons |   4.991105   .5783784     8.63   0.000      3.75832    6.223889
------------------------------------------------------------------------------

  4 . predict d, cooksd

  5 . sort d

  6 . br country d

  7 .
end of do-file

  8 . do "C:\Users\HP\AppData\Local\Temp\STD31f4_000000.tmp"

  9 . predict diffits, dfits

 10 . generate absdiffits=abs(diffits)

 11 . sort absdiffits
```

```
12 . br country absdiffits

13 .
   end of do-file

14 . do "C:\Users\HP\AppData\Local\Temp\STD31f4_000000.tmp"

15 . drop in 15/17
   (3 observations deleted)

16 .
   end of do-file

17 . do "C:\Users\HP\AppData\Local\Temp\STD31f4_000000.tmp"

18 . reg lny1 temp1

      Source |       SS           df       MS      Number of obs   =        14
-------------+----------------------------------   F(1, 12)        =      2.45
       Model |  1.64228679         1  1.64228679   Prob > F        =    0.1438
    Residual |  8.05771321        12  .671476101   R-squared       =    0.1693
-------------+----------------------------------   Adj R-squared   =    0.1001
       Total |         9.7        13  .746153846   Root MSE        =    .81944

------------------------------------------------------------------------------
        lny1 |      Coef.   Std. Err.      t    P>|t|     [95% Conf. Interval]
-------------+----------------------------------------------------------------
       temp1 |  -.0554827   .0354771    -1.56   0.144    -.1327806    .0218153
       _cons |   4.993533   .4945934    10.10   0.000     3.915907     6.07116
------------------------------------------------------------------------------

19 .
   end of do-file

20 . import excel "E:\ا نتا ج علمى\IN PROGRESS\انوروك \Analysis\results\STATA 16.1\B
   > ook1.xlsx", sheet("March") firstrow clear
   (9 vars, 17 obs)

21 . do "C:\Users\HP\AppData\Local\Temp\STD31f4_000000.tmp"

22 . reg lny1 temp1

      Source |       SS           df       MS      Number of obs   =        17
-------------+----------------------------------   F(1, 15)        =      2.30
       Model |  2.90288281         1  2.90288281   Prob > F        =    0.1503
    Residual |  18.9418231        15   1.2627882   R-squared       =    0.1329
-------------+----------------------------------   Adj R-squared   =    0.0751
       Total |  21.8447059        16  1.36529412   Root MSE        =    1.1237

------------------------------------------------------------------------------
        lny1 |      Coef.   Std. Err.      t    P>|t|     [95% Conf. Interval]
-------------+----------------------------------------------------------------
       temp1 |  -.0586999   .0387158    -1.52   0.150    -.1412206    .0238208
       _cons |   4.991105   .5783784     8.63   0.000      3.75832    6.223889
------------------------------------------------------------------------------

23 . predict d, cooksd

24 . sort d
```

```
25 . br country d

26 .
   end of do-file

27 . do "C:\Users\HP\AppData\Local\Temp\STD31f4_000000.tmp"

28 . predict diffits, dfits

29 . generate absdiffits=abs(diffits)

30 . sort absdiffits

31 . br country absdiffits

32 .
   end of do-file

33 . do "C:\Users\HP\AppData\Local\Temp\STD31f4_000000.tmp"

34 . drop in 15/17
   (3 observations deleted)

35 . reg lny1 temp1

         Source |       SS           df       MS      Number of obs   =        14
   -------------+----------------------------------   F(1, 12)        =      2.45
          Model |  1.64228679         1  1.64228679   Prob > F        =    0.1438
       Residual |  8.05771321        12  .671476101   R-squared       =    0.1693
   -------------+----------------------------------   Adj R-squared   =    0.1001
          Total |         9.7        13  .746153846   Root MSE        =    .81944

   ------------------------------------------------------------------------------
           lny1 |      Coef.   Std. Err.      t    P>|t|     [95% Conf. Interval]
   -------------+----------------------------------------------------------------
          temp1 |  -.0554827   .0354771    -1.56   0.144    -.1327806    .0218153
          _cons |   4.993533   .4945934    10.10   0.000     3.915907     6.07116
   ------------------------------------------------------------------------------

36 .
   end of do-file

37 . import excel "E:\ا نتا ج علمی\IN PROGRESS\انوروک \Analysis\results\STATA 16.1\Book1.xlsx", sheet("March")
   > firstrow clear
   (9 vars, 17 obs)

38 . do "C:\Users\HP\AppData\Local\Temp\STD31f4_000000.tmp"

39 . reg lny2 temp2

         Source |       SS           df       MS      Number of obs   =        17
   -------------+----------------------------------   F(1, 15)        =      1.97
          Model |  2.50632354         1  2.50632354   Prob > F        =    0.1809
       Residual |  19.0913235        15   1.2727549   R-squared       =    0.1160
   -------------+----------------------------------   Adj R-squared   =    0.0571
          Total |  21.5976471        16  1.34985294   Root MSE        =    1.1282

   ------------------------------------------------------------------------------
           lny2 |      Coef.   Std. Err.      t    P>|t|     [95% Conf. Interval]
   -------------+----------------------------------------------------------------
          temp2 |  -.0638841   .0455246    -1.40   0.181    -.1609176    .0331494
          _cons |   6.232447   .7108605     8.77   0.000     4.717284    7.747611
   ------------------------------------------------------------------------------
```

```
40 . predict d, cooksd

41 . sort d

42 . br country d

43 .
   end of do-file

44 . do "C:\Users\HP\AppData\Local\Temp\STD31f4_000000.tmp"

45 . predict diffits, dfits

46 . generate absdiffits=abs(diffits)

47 . sort absdiffits

48 . br country absdiffits

49 .
   end of do-file

50 . do "C:\Users\HP\AppData\Local\Temp\STD31f4_000000.tmp"

51 . drop in 16/17
   (2 observations deleted)

52 . reg lny2 temp2
```

Source	SS	df	MS			
				Number of obs	=	15
				F(1, 13)	=	0.39
Model	.35042327	1	.35042327	Prob > F	=	0.5412
Residual	11.5669101	13	.889762313	R-squared	=	0.0294
				Adj R-squared	=	-0.0453
Total	11.9173333	14	.851238095	Root MSE	=	.94327

lny2	Coef.	Std. Err.	t	P>|t|	[95% Conf.	Interval]
temp2	-.0276577	.0440714	-0.63	0.541	-.1228682	.0675528
_cons	5.689478	.6469646	8.79	0.000	4.291796	7.08716

```
53 .
   end of do-file

54 . import excel "E:\ا نتا ج علمى\IN PROGRESS\كورونا \Analysis\results\STATA 16.1\Book1.xlsx", sheet("March")
   > firstrow clear
   (9 vars, 17 obs)

55 . do "C:\Users\HP\AppData\Local\Temp\STD31f4_000000.tmp"

56 . reg lny3 temp3
```

Source	SS	df	MS			
				Number of obs	=	17
				F(1, 15)	=	0.01
Model	.022247111	1	.022247111	Prob > F	=	0.9064
Residual	23.3565764	15	1.55710509	R-squared	=	0.0010
				Adj R-squared	=	-0.0657
Total	23.3788235	16	1.46117647	Root MSE	=	1.2478

lny3	Coef.	Std. Err.	t	P>|t|	[95% Conf.	Interval]
temp3	-.0066667	.055774	-0.12	0.906	-.1255462	.1122128
_cons	6.439373	.9218278	6.99	0.000	4.474543	8.404202

```
57 . predict d, cooksd

58 . sort d

59 . br country d

60 .
   end of do-file

61 . do "C:\Users\HP\AppData\Local\Temp\STD31f4_000000.tmp"

62 . predict diffits, dfits

63 . generate absdiffits=abs(diffits)

64 . sort absdiffits

65 . br country absdiffits

66 .
   end of do-file

67 . do "C:\Users\HP\AppData\Local\Temp\STD31f4_000000.tmp"

68 . drop in 14/17
   (4 observations deleted)

69 . reg lny3 temp3

      Source |       SS           df       MS      Number of obs   =        13
-------------+----------------------------------   F(1, 11)        =      0.19
       Model |  .213534009         1  .213534009   Prob > F        =    0.6701
    Residual |  12.2587737        11  1.11443397   R-squared       =    0.0171
-------------+----------------------------------   Adj R-squared   =   -0.0722
       Total |  12.4723077        12  1.03935897   Root MSE        =    1.0557

------------------------------------------------------------------------------
        lny3 |      Coef.   Std. Err.      t    P>|t|     [95% Conf. Interval]
-------------+----------------------------------------------------------------
       temp3 |  -.0451153   .1030664    -0.44   0.670    -.2719629    .1817324
       _cons |   7.043411   1.395501     5.05   0.000     3.971933    10.11489
------------------------------------------------------------------------------

70 .
   end of do-file

71 . import excel "E:\انتاج علمى\IN PROGRESS\كورونا \Analysis\results\STATA 16.1\Book1.xlsx", sheet("March")
   > firstrow clear
   (9 vars, 17 obs)

72 . do "C:\Users\HP\AppData\Local\Temp\STD31f4_000000.tmp"

73 . reg lny4 temp4

      Source |       SS           df       MS      Number of obs   =        17
-------------+----------------------------------   F(1, 15)        =      0.02
       Model |  .043406611         1  .043406611   Prob > F        =    0.8917
    Residual |  33.9754169        15  2.26502779   R-squared       =    0.0013
-------------+----------------------------------   Adj R-squared   =   -0.0653
       Total |  34.0188235        16  2.12617647   Root MSE        =     1.505

------------------------------------------------------------------------------
        lny4 |      Coef.   Std. Err.      t    P>|t|     [95% Conf. Interval]
-------------+----------------------------------------------------------------
       temp4 |  -.0108837   .0786202    -0.14   0.892    -.1784586    .1566912
       _cons |   7.362276   1.473126     5.00   0.000     4.222383    10.50217
------------------------------------------------------------------------------
```

```
74 . predict d, cooksd

75 . sort d

76 . br country d

77 .
   end of do-file

78 . do "C:\Users\HP\AppData\Local\Temp\STD31f4_000000.tmp"

79 . predict diffits, dfits

80 . generate absdiffits=abs(diffits)

81 . sort absdiffits

82 . br country absdiffits

83 .
   end of do-file

84 . do "C:\Users\HP\AppData\Local\Temp\STD31f4_000000.tmp"

85 . drop in 14/17
   (4 observations deleted)

86 . reg lny4 temp4

      Source |       SS           df       MS      Number of obs   =        13
-------------+----------------------------------   F(1, 11)        =      0.00
       Model |  .001661973         1  .001661973   Prob > F        =    0.9702
    Residual |  12.5091073        11  1.13719157   R-squared       =    0.0001
-------------+----------------------------------   Adj R-squared   =   -0.0908
       Total |  12.5107692        12   1.0425641   Root MSE        =    1.0664

------------------------------------------------------------------------------
        lny4 |      Coef.   Std. Err.      t    P>|t|     [95% Conf. Interval]
-------------+----------------------------------------------------------------
       temp4 |  -.0046564   .1218019    -0.04   0.970    -.2727406    .2634279
       _cons |   7.317405   2.086084     3.51   0.005     2.725965    11.90885
------------------------------------------------------------------------------

87 .
   end of do-file

88 .
```
