# Supplementary material for: Does Temperature Affect COVID-19 Transmission?
Source: Front Public Health. 2020 Dec 22;8:554964. doi: 10.3389/fpubh.2020.554964 (PMC7793668; doi:10.3389/fpubh.2020.554964)
Supplement: Supplementary file 5 [file Data_Sheet_5.PDF]

```
  ___  ____  ____  ____  ____(R)
 /__    /   ____/   /   ____/
___/   /   /___/   /   /___/
  Statistics/Data analysis
```

Project: Does Weather Temperature affect COVID-19 Transmission?

```
  ___  ____  ____  ____  ____ (R)
 /__    /   ____/   /   ____/
___/   /   /___/   /   /___/   16.1   Copyright 1985-2019 StataCorp LLC
  Statistics/Data analysis            StataCorp
                                      4905 Lakeway Drive
     MP - Parallel Edition            College Station, Texas 77845 USA
                                      800-STATA-PC        https://www.stata.com
                                      979-696-4600        stata@stata.com
                                      979-696-4601 (fax)

Stata license: Single-user 2-core network, expiring 17 Sep 2020
Serial number: 501609327014
  Licensed to:


Notes:
      1. Unicode is supported; see help unicode_advice.
      2. More than 2 billion observations are allowed; see help obs_advice.
      3. Maximum number of variables is set to 5,000; see help set_maxvar.
      4. New update available; type -update all-

  1 . import excel "E:\انتاج علمى\IN PROGRESS\كورونا \Analysis\results\STATA 16.1\B
    > ook1.xlsx", sheet("March with Togo") firstrow
    (9 vars, 18 obs)

  2 . do "C:\Users\HP\AppData\Local\Temp\STD2bb4_000000.tmp"

  3 . reg lny1 temp1
```

Source	SS	df	MS			
				Number of obs	=	**18**
				F(1, 16)	=	**2.68**
Model	**5.00416538**	**1**	**5.00416538**	Prob > F	=	**0.1208**
Residual	**29.8246791**	**16**	**1.86404244**	R-squared	=	**0.1437**
				Adj R-squared	=	**0.0902**
Total	**34.8288444**	**17**	**2.04875556**	Root MSE	=	**1.3653**

lny1	Coef.	Std. Err.	t	P>|t|	[95% Conf.	Interval]
temp1	**-.0680068**	**.0415063**	**-1.64**	**0.121**	**-.1559963**	**.0199827**
_cons	**4.811877**	**.6885826**	**6.99**	**0.000**	**3.352147**	**6.271607**

```
  4 . predict d, cooksd

  5 . sort d

  6 . br country d

  7 .
    end of do-file

  8 . do "C:\Users\HP\AppData\Local\Temp\STD2bb4_000000.tmp"

  9 . predict diffits, dfits

 10 . generate absdiffits=abs(diffits)

 11 . sort absdiffits
```

12 . br country absdiffits

13 .
end of do-file

14 . do "C:\Users\HP\AppData\Local\Temp\STD2bb4_000000.tmp"

15 . drop in 14/18
(5 observations deleted)

16 .
end of do-file

17 . do "C:\Users\HP\AppData\Local\Temp\STD2bb4_000000.tmp"

18 . reg lny1 temp1

Source	SS	df	MS			
				Number of obs	=	13
				F(1, 11)	=	7.12
Model	4.91865664	1	4.91865664	Prob > F	=	0.0219
Residual	7.60083567	11	.690985061	R-squared	=	0.3929
				Adj R-squared	=	0.3377
Total	12.5194923	12	1.04329103	Root MSE	=	.83126

lny1	Coef.	Std. Err.	t	P>|t|	[95% Conf.	Interval]
temp1	-.0852226	.0319423	-2.67	0.022	-.1555271	-.0149181
_cons	5.124552	.4900665	10.46	0.000	4.045923	6.203182

19 .
end of do-file

20 . import excel "E:\ا نتا ج علمى\IN PROGRESS\كورونا \Analysis\results\STATA 16.1\B
> ook1.xlsx", sheet("March with Togo") firstrow clear
(9 vars, 18 obs)

21 . do "C:\Users\HP\AppData\Local\Temp\STD2bb4_000000.tmp"

22 . reg lny2 temp2

Source	SS	df	MS			
				Number of obs	=	18
				F(1, 16)	=	4.92
Model	6.97655647	1	6.97655647	Prob > F	=	0.0414
Residual	22.6966213	16	1.41853883	R-squared	=	0.2351
				Adj R-squared	=	0.1873
Total	29.6731778	17	1.74548105	Root MSE	=	1.191

lny2	Coef.	Std. Err.	t	P>|t|	[95% Conf.	Interval]
temp2	-.0960488	.0433104	-2.22	0.041	-.1878627	-.0042349
_cons	6.523001	.7465545	8.74	0.000	4.940377	8.105626

23 . predict d, cooksd

24 . sort d

```
25 . br country d

26 .
   end of do-file

27 . do "C:\Users\HP\AppData\Local\Temp\STD2bb4_000000.tmp"

28 . predict diffits, dfits

29 . generate absdiffits=abs(diffits)

30 . sort absdiffits

31 . br country absdiffits

32 .
   end of do-file

33 . do "C:\Users\HP\AppData\Local\Temp\STD2bb4_000000.tmp"

34 . drop in 14/18
   (5 observations deleted)

35 .
   end of do-file

36 . do "C:\Users\HP\AppData\Local\Temp\STD2bb4_000000.tmp"

37 . reg lny2 temp2

         Source |       SS           df       MS      Number of obs   =        13
   -------------+----------------------------------   F(1, 11)        =      8.54
          Model |  6.72345053         1  6.72345053   Prob > F        =    0.0139
       Residual |  8.65645716        11  .786950651   R-squared       =    0.4372
   -------------+----------------------------------   Adj R-squared   =    0.3860
          Total |  15.3799077        12  1.28165897   Root MSE        =     .8871

   ------------------------------------------------------------------------------
           lny2 |      Coef.   Std. Err.      t    P>|t|     [95% Conf. Interval]
   -------------+----------------------------------------------------------------
          temp2 |  -.1511554   .0517132    -2.92   0.014    -.2649754   -.0373354
          _cons |   7.146531   .7797316     9.17   0.000     5.430354    8.862709
   ------------------------------------------------------------------------------

38 .
   end of do-file

39 . import excel "E:\ا نتا ج علمی\IN PROGRESS\انوروك \Analysis\results\STATA 16.1\B
   > ook1.xlsx", sheet("March with Togo") firstrow clear
   (9 vars, 18 obs)

40 . do "C:\Users\HP\AppData\Local\Temp\STD2bb4_000000.tmp"

41 . reg lny3 temp3

         Source |       SS           df       MS      Number of obs   =        18
   -------------+----------------------------------   F(1, 16)        =      0.97
          Model |  1.75901362         1  1.75901362   Prob > F        =    0.3384
       Residual |  28.9024808        16  1.80640505   R-squared       =    0.0574
   -------------+----------------------------------   Adj R-squared   =   -0.0015
          Total |  30.6614944        17  1.80361732   Root MSE        =     1.344

   ------------------------------------------------------------------------------
           lny3 |      Coef.   Std. Err.      t    P>|t|     [95% Conf. Interval]
   -------------+----------------------------------------------------------------
          temp3 |  -.0536925    .054411    -0.99   0.338    -.1690386    .0616536
          _cons |   6.947444   .9731556     7.14   0.000     4.884446    9.010442
   ------------------------------------------------------------------------------
```

```
42 . predict d, cooksd

43 . sort d

44 . br country d

45 .
   end of do-file

46 . do "C:\Users\HP\AppData\Local\Temp\STD2bb4_000000.tmp"

47 . predict diffits, dfits

48 . generate absdiffits=abs(diffits)

49 . sort absdiffits

50 . br country absdiffits

51 .
   end of do-file

52 . do "C:\Users\HP\AppData\Local\Temp\STD2bb4_000000.tmp"

53 . drop in 16/18
   (3 observations deleted)

54 .
   end of do-file

55 . do "C:\Users\HP\AppData\Local\Temp\STD2bb4_000000.tmp"

56 . reg lny3 temp3

         Source |       SS           df       MS      Number of obs   =        15
   -------------+----------------------------------   F(1, 13)        =      2.58
          Model |  3.13643527         1  3.13643527   Prob > F        =    0.1325
       Residual |  15.8280581        13  1.21754293   R-squared       =    0.1654
   -------------+----------------------------------   Adj R-squared   =    0.1012
          Total |  18.9644933        14  1.35460667   Root MSE        =    1.1034

   ------------------------------------------------------------------------------
           lny3 |      Coef.   Std. Err.      t    P>|t|     [95% Conf. Interval]
   -------------+----------------------------------------------------------------
          temp3 |  -.1198775   .0746899    -1.61   0.133    -.2812352    .0414801
          _cons |   7.809504   1.151181     6.78   0.000      5.32253    10.29648
   ------------------------------------------------------------------------------

57 .
   end of do-file

58 . import excel "E:\انتاج علمى\IN PROGRESS\كورونا \Analysis\results\STATA 16.1\B
   > ook1.xlsx", sheet("March with Togo") firstrow clear
   (9 vars, 18 obs)

59 . do "C:\Users\HP\AppData\Local\Temp\STD2bb4_000000.tmp"

60 . reg lny4 temp4

         Source |       SS           df       MS      Number of obs   =        18
   -------------+----------------------------------   F(1, 16)        =      0.67
          Model |  1.69381583         1  1.69381583   Prob > F        =    0.4235
       Residual |  40.1640953        16  2.51025596   R-squared       =    0.0405
   -------------+----------------------------------   Adj R-squared   =   -0.0195
          Total |  41.8579111        17  2.46223007   Root MSE        =    1.5844
```

lny4	Coef.	Std. Err.	t	P>|t|	[95% Conf.	Interval]
temp4	**-.0621128**	**.0756148**	**-0.82**	**0.423**	**-.2224091**	**.0981835**
_cons	**8.199943**	**1.469792**	**5.58**	**0.000**	**5.084123**	**11.31576**

```
61 . predict d, cooksd

62 . sort d

63 . br country d

64 .
   end of do-file

65 . do "C:\Users\HP\AppData\Local\Temp\STD2bb4_000000.tmp"

66 . predict diffits, dfits

67 . generate absdiffits=abs(diffits)

68 . sort absdiffits

69 . br country absdiffits

70 .
   end of do-file

71 . do "C:\Users\HP\AppData\Local\Temp\STD2bb4_000000.tmp"

72 . predict diffits, dfits
   variable diffits already defined
   r(110);

   end of do-file

   r(110);

73 . do "C:\Users\HP\AppData\Local\Temp\STD2bb4_000000.tmp"

74 . predict diffits, dfits
   variable diffits already defined
   r(110);

   end of do-file

   r(110);

75 . do "C:\Users\HP\AppData\Local\Temp\STD2bb4_000000.tmp"

76 . predict diffits, dfits
   variable diffits already defined
   r(110);

   end of do-file

   r(110);

77 . do "C:\Users\HP\AppData\Local\Temp\STD2bb4_000000.tmp"
```

```
78 . predict diffits, dfits
   variable diffits already defined
   r(110);

   end of do-file

   r(110);

79 . do "C:\Users\HP\AppData\Local\Temp\STD2bb4_000000.tmp"

80 . predict diffits, dfits
   variable diffits already defined
   r(110);

   end of do-file

   r(110);

81 . do "C:\Users\HP\AppData\Local\Temp\STD2bb4_000000.tmp"

82 . predict diffits, dfits
   variable diffits already defined
   r(110);

   end of do-file

   r(110);

83 . do "C:\Users\HP\AppData\Local\Temp\STD2bb4_000000.tmp"

84 . predict diffits, dfits
   variable diffits already defined
   r(110);

   end of do-file

   r(110);

85 . do "C:\Users\HP\AppData\Local\Temp\STD2bb4_000000.tmp"

86 . predict diffits, dfits
   variable diffits already defined
   r(110);

   end of do-file

   r(110);

87 . do "C:\Users\HP\AppData\Local\Temp\STD2bb4_000000.tmp"

88 . drop in 16/18
   (3 observations deleted)

89 .
   end of do-file

90 . do "C:\Users\HP\AppData\Local\Temp\STD2bb4_000000.tmp"

91 . reg lny4 temp4
```

Source	SS	df	MS			
				Number of obs	=	15
				F(1, 13)	=	1.64
Model	2.80106741	1	2.80106741	Prob > F	=	0.2228
Residual	22.2134259	13	1.70872507	R-squared	=	0.1120
				Adj R-squared	=	0.0437
Total	25.0144933	14	1.78674952	Root MSE	=	1.3072

lny4	Coef.	Std. Err.	t	P>|t|	[95% Conf.	Interval]
temp4	**-.1745648**	**.1363424**	**-1.28**	**0.223**	**-.4691148**	**.1199851**
_cons	**9.989674**	**2.423247**	**4.12**	**0.001**	**4.754568**	**15.22478**

```
92 .
   end of do-file

93 .
```
